# Parallel Implementation of Large-Scale Linear Scaling Density Functional Theory Calculations With Numerical Atomic Orbitals in HONPAS

**DOI:** 10.3389/fchem.2020.589910

**Published:** 2020-11-26

**Authors:** Zhaolong Luo, Xinming Qin, Lingyun Wan, Wei Hu, Jinlong Yang

**Affiliations:** Hefei National Laboratory for Physical Sciences at the Microscale, Department of Chemical Physics, and Synergetic Innovation Center of Quantum Information and Quantum Physics, University of Science and Technology of China, Hefei, China

**Keywords:** linear-scaling density functional theory, density matrix purification algorithm, sparse matrix multiplication, parallel implementation, tens of thousands of atoms

## Abstract

Linear-scaling density functional theory (DFT) is an efficient method to describe the electronic structures of molecules, semiconductors, and insulators to avoid the high cubic-scaling cost in conventional DFT calculations. Here, we present a parallel implementation of linear-scaling density matrix trace correcting (TC) purification algorithm to solve the Kohn–Sham (KS) equations with the numerical atomic orbitals in the HONPAS package. Such a linear-scaling density matrix purification algorithm is based on the Kohn's nearsightedness principle, resulting in a sparse Hamiltonian matrix with localized basis sets in the DFT calculations. Therefore, sparse matrix multiplication is the most time-consuming step in the density matrix purification algorithm for linear-scaling DFT calculations. We propose to use the MPI_Allgather function for parallel programming to deal with the sparse matrix multiplication within the compressed sparse row (CSR) format, which can scale up to hundreds of processing cores on modern heterogeneous supercomputers. We demonstrate the computational accuracy and efficiency of this parallel density matrix purification algorithm by performing large-scale DFT calculations on boron nitrogen nanotubes containing tens of thousands of atoms.

## 1. Introduction

The Kohn–Sham density functional theory (DFT) (Hohenberg and Kohn, 1964; Kohn and Sham, 1965) has been successfully applied to perform first-principles calculations for describing the electronic structures of both molecules and solids. However, conventional DFT calculations based on direct diagonalization methods for solving the KS equations have a high cubic-scaling cost (Goedecker, 1999), which can usually be used to study medium-scale systems containing up to hundreds of atoms. Therefore, it is difficult to achieve massive parallelism for these conventional cubic-scaling methods due to complex communication issues. To avoid the bottleneck arising from the computational cost and memory usage of directly diagonalizing the Hamiltonian matrix in conventional DFT calculations, linear-scaling methods using local basis functions have been proposed (Goedecker, 1999), strongly promoting the applications of DFT calculations in large-scale systems containing thousands of atoms.

In general, linear-scaling methods include direct, variational, and purification methods (Bowler and Miyazaki, 2012). The direct methods are featured by direct evaluation of density matrix using various approximations, including divide and conquer (Yang, 1991; Yang and Lee, 1995) and Fermi operator expansion (Goedecker and Colombo, 1994; Goedecker and Teter, 1995; Liang et al., 2003). The variational methods minimize the total energy with respect to the auxiliary density matrix or Wannier-like orbitals, covering density matrix minimization method (Daw, 1993; Li et al., 1993; Nunes and Vanderbilt, 1994) and orbital minimization method (OMM) (Galli and Parrinello, 1992; Mauri and Galli, 1994; Kim et al., 1995; Ordejón et al., 1995). The third scheme exploits the purification polynomial and iterative solution, which is known as density matrix purification method (Palser and Manolopoulos, 1998; Niklasson, 2002; Niklasson et al., 2003). Nearly all of the linear-scaling methods are based on the Kohn's nearsightedness principle with localized basis sets, such as Gaussian type orbitals (GTOs) (Frisch et al., 1984) and numerical atomic orbitals (Shang et al., 2010) (NAOs), resulting in the sparsity of density matrix with a number of non-zero entries that increase linearly with the system size, so the linear-scaling matrix-matrix multiplication can be achieved (VandeVondele et al., 2012; Kim and Jung, 2016). In particular, the density matrix purification algorithms without prior knowledge of the chemical potential, including the trace-preserving canonical purification scheme of Palser and Manolopoulos (PM) (Palser and Manolopoulos, 1998; Daniels and Scuseria, 1999), the trace-correcting purification (TC) (Niklasson, 2002), and the trace resetting density matrix purification (TRS) (Niklasson et al., 2003), have been demonstrated as accurate and efficient linear-scaling methods to describe the electronic structures of molecules, semiconductors, and insulators. However, almost all of the developed linear-scaling techniques (direct, variational, and purification methods) assume the presence of a non-zero gap in the electronic structure, which prevents them from treating metallic systems. Recently, Suryanarayana (2017) have employed the *O*(*N*) Spectral Quadrature (SQ) method (Suryanarayana, 2013; Pratapa et al., 2016) to study the locality of electronic interactions in aluminum (a prototypical metallic system) as a function of smearing/electronic temperature. They have found exponential convergence accompanied by a rate that increases sub-linearly with smearing. It is also worth mentioning that all these linear-scaling methods based on Kohn's nearsightedness principle are limited to the localization of density matrix (Bowler and Miyazaki, 2012). A recently published innovative version of PEXSI scheme named iPEXSI (Etter, 2020), which does not rely on the nearsightedness principle, can scale provably better than cubically even in the absence of localization of density matrix. The iPEXSI algorithm utilizes a localization property of triangular factorization, which could extend the usable range of linear-scaling method to metallic system without the constraint of finite electronic temperature.

Nowadays, with the rapid development of modern heterogeneous supercomputers, the high-performance computing (HPC) has become a powerful tool for accelerating the DFT calculations to deal with large-scale systems. Several highly efficient DFT software based on low-scaling methods have been developed, such as SIESTA (Soler et al., 2002), OPENMX (Ozaki and Kino, 2005), CP2K (Kühne et al., 2020), CONQUEST (Gillan et al., 2007), PROFESS (Ho et al., 2008), FREEON (Challacombe, 2014), ONETEP (Skylaris et al., 2005), BigDFT (Genovese et al., 2008; Mohr et al., 2014), FHI-aims (Blum et al., 2009), ABACUS (Chen et al., 2010, 2011), HONPAS (Qin et al., 2015), and DGDFT (Lin et al., 2012; Hu et al., 2015a,b; Banerjee et al., 2016; Zhang et al., 2017), which are capable to make full advantage of the massive parallelism available on HPC architectures beneting from the local data communication of sparse Hamiltonian matrix generated with local basis sets. In linear-scaling DFT calculations, the kernel for HPC is to parallel sparse matrix–matrix multiplication. In order to realize the HPC parallelism, two massively parallel libraries of BCSR (Borštnik et al., 2014) and NTPOLY (Dawson and Nakajima, 2018) have been developed, which have shown a high performance for the density matrix purification algorithms implemented in the CP2K (Kühne et al., 2020) and CONQUEST (Gillan et al., 2007) packages.

In this work, we present a parallel implementation of linear-scaling density matrix second-order trace-correcting purification (TC2) algorithm (Niklasson, 2002) to solve the KS equations with the NAOs in the HONPAS package (Qin et al., 2015). We propose to use the MPI_Allgather function for parallel programming to deal with such sparse matrix multiplication within the CSR format, which can be scaled linearly up to hundreds of processing cores on modern heterogeneous supercomputers. We demonstrate the computational accuracy and efficiency of this linear-scaling density matrix purification method by performing large-scale DFT calculations on boron nitrogen nanotubes containing thousands of atoms.

## 2. Methodology

### 2.1. Density Functional Theory

We first give a brief review of Kohn–Sham density functional theory (KS-DFT). The key spirit of KS-DFT is to solve the KS equations defined as

(1) $$\begin{array}{l} \hat{H}\psi_{i}\left(\text{r}\right)=\left(\hat{T}+{\hat{V}}_{\text{ion}}+{\hat{V}}_{\text{H}}+{\hat{V}}_{\text{xc}}\right)\psi_{i}\left(\text{r}\right)=\varepsilon_{i}\psi_{i}\left(\text{r}\right) \end{array}$$

where $\hat{H}$ is the Hamiltonian operator, ψ_*i*_ is the *i*th molecular orbital, and ϵ_*i*_ is the corresponding orbital energy. $\hat{T}$ is the kinetic operator, ${\hat{V}}_{\text{ion}}$ is the ionic potential operator, and ${\hat{V}}_{\text{H}}$ is the Hartree potential operator defined as

(2) $$\begin{array}{l} {\hat{V}}_{\text{H}}\left(\text{r}\right)=\int \frac{\rho \left({\text{r}}^{\prime }\right)}{\left|\text{r}-{\text{r}}^{\prime }\right|}d{\text{r}}^{\prime } \end{array}$$

where the electron density is given by

(3) $$\begin{array}{l} \rho \left(\text{r}\right)=\overset{N_{e}}{\underset{i=1}{\sum }}\psi_{i}^{*}\left(\text{r}\right)\psi_{i}\left(\text{r}\right) \end{array}$$

In the approximation of linear combination of atomic orbitals (LCAO) (Mulliken, 1955), the ψ_*i*_ is expanded on a set of NAOs ${\left\{\phi_{\mu }\left(r\right)\right\}}_{\mu =1}^{N_{\text{b}}}$

(4) $$\begin{array}{l} \psi_{i}\left(\text{r}\right)=\overset{N_{\text{b}}}{\underset{\mu }{\sum }}c_{\mu i}\phi_{\mu }\left(\text{r}\right) \end{array}$$

where *c*_μ*i*_ is the expansion coefficient at the μth atomic orbital and *N*_b_ is the number of NAOs. Then, the KS equations can be rewritten into matrix notations as

(5) $$\begin{array}{l} HC=SCE \end{array}$$

where *C* is coefficient matrix and *E* is the corresponding orbital energy. *H* and *S* are the Hamiltonian and overlap matrices over the NAOs

(6) $$\begin{array}{c} H_{\mu \nu }=\int \phi_{\mu }^{*}\left(\text{r}\right)\hat{H}\phi_{\nu }\left(\text{r}\right)d\text{r}\\ \;S_{\mu \nu }=\int \phi_{\mu }^{*}\left(\text{r}\right)\phi_{\nu }\left(\text{r}\right)d\text{r} \end{array}$$

The default choice in the SIESTA package is to use the direct diagonalization method though the LAPACK and ScaLAPACK libraries to solve this eigenvalue problem with a high cubic-scaling cost. Therefore, the computational cost and memory usage of such DFT calculations increase rapidly as the system size, which are only limited to small systems containing hundreds of atoms. In order to overcome this limitation, several linear-scaling methods have been implemented in the SIESTA package, such as the Kim–Mauri–Galli (KMG) orbital minimization (OMM) method (Galli and Parrinello, 1992; Mauri and Galli, 1994; Kim et al., 1995; Corsetti, 2014) and divide and conquer method (Cankurtaran et al., 2008). The KMG requires a initial approximate Wannier functions and a prior knowledge of the chemical potential. In the HONPAS-SIESTA package (Qin et al., 2015), we implement the density matrix purification algorithms, including the trace-preserving canonical purification scheme of PM (Palser and Manolopoulos, 1998; Daniels and Scuseria, 1999), the trace-correcting purification (TC) (Niklasson, 2002), and the trace resetting density matrix purification (TRS) (Niklasson et al., 2003).

### 2.2. Linear-Scaling Density Matrix Purification Algorithms

After constructing the Hamiltonian matrix, the density matrix can be obtained by directly diagonalizing the Hamiltonian matrix with cubic-scaling cost. In order to avoid the high cost of explicit diagonalization, we implement three density matrix purification algorithms, without prior knowledge of the chemical potential for linear-scaling DFT calculations, including the trace-preserving canonical purification scheme of PM, the trace-correcting purification (TC) (Niklasson, 2002), and the trace resetting density matrix purification (TRS) (Niklasson et al., 2003), in the HONPAS package (Qin et al., 2015). In this work, we use the second-order trace-correcting purification (TC2) (Niklasson, 2002) algorithm with orthogonal basis sets to illustrate our parallel algorithms. In the coordinate presentation, the general form of density matrix can be given by

(7) $$\rho (\text{r},{\text{r}}^{\prime })=\sum_{i=1}^{N_{b}}f(\varepsilon_{i})\psi_{i}(\text{r})\psi_{i}^{*}({\text{r}}^{\prime })$$

where *f*(ε_*i*_) is the Fermi distribution function of energy level ε_*i*_ at finite electronic temperature

(8) $$\begin{array}{l} f\left(\varepsilon_{i}\right)=\frac{1}{1+e^{\beta \left(\varepsilon_{i}-\mu \right)}} \end{array}$$

with the chemical potential μ and the inverse temperature β = 1/*k*_*B*_*T*. Within the LCAO method, we can transform the density matrix from coordinate presentation to the basis presentation, then the density matrix element *P*_μν_ becomes:

(9) $$\begin{array}{l} P_{\mu \nu }=\int \phi_{\mu }^{*}(\text{r})\rho (\text{r},{\text{r}}^{\prime })\phi_{\nu }({\text{r}}^{\prime })d\text{r}d{\text{r}}^{\prime }\\ \;=\int \phi_{\mu }^{*}(\text{r})\sum_{i=1}^{N_{\text{b}}}f(\varepsilon_{i})\psi_{i}(\text{r})\psi_{i}^{*}({\text{r}}^{\prime })\phi_{\nu }({\text{r}}^{\prime })d\text{r}d{\text{r}}^{\prime }\\ \;=\sum_{i=1}^{N_{b}}f(\varepsilon_{i})\sum_{\lambda }^{N_{\text{b}}}c_{\lambda i}\sum_{\kappa }^{N_{\text{b}}}c_{\kappa i}^{*}\int \phi_{\mu }^{*}(\text{r})\phi_{\lambda }(\text{r})d\text{r}\int \phi_{\kappa }^{*}({\text{r}}^{\prime })\phi_{\nu }({\text{r}}^{\prime })d{\text{r}}^{\prime }\\ \;=\sum_{i=1}^{N_{b}}f(\varepsilon_{i})\sum_{\lambda }^{N_{\text{b}}}c_{\lambda i}\sum_{\kappa }^{N_{\text{b}}}c_{\kappa i}^{*}S_{\mu \lambda }S_{\nu \kappa } \end{array}$$

If NAOs are orthogonal, the density matrix element *P*_μν_ can be written as

(10) $$\begin{array}{l} P_{\mu \nu }=\overset{N_{b}}{\underset{i}{\sum }}f\left(\varepsilon_{i}\right)c_{\mu i}c_{\nu i}^{*} \end{array}$$

Note that ε_*i*_ is relative to the eigenvalue of $\hat{H}$ψ_*i*_ = ε_*i*_ψ_*i*_, so *P*_μν_ can be rewritten as

(11) $$\begin{array}{l} P_{\mu \nu }=\int \phi_{\mu }^{*}(\text{r})\rho (\text{r},{\text{r}}^{\prime })\phi_{\nu }({\text{r}}^{\prime })d\text{r}d{\text{r}}^{\prime }\\ \;=\sum_{i}^{N_{b}}\int \phi_{\mu }^{*}(\text{r})f(\varepsilon_{i})\psi_{i}(\text{r})\psi_{i}^{*}({\text{r}}^{\prime })\phi_{\nu }({\text{r}}^{\prime })d\text{r}d{\text{r}}^{\prime }\\ \;=\int \phi_{\mu }^{*}(\text{r})f(\hat{H})\sum_{i}^{N_{b}}\left(\psi_{i}(\text{r})\psi_{i}^{*}({\text{r}}^{\prime })\right)\phi_{\nu }({\text{r}}^{\prime })d\text{r}d{\text{r}}^{\prime }\\ \;=\int \phi_{\mu }^{*}(\text{r})f(\hat{H})\phi_{\nu }({\text{r}}^{\prime })d\text{r}d{\text{r}}^{\prime } \end{array}$$

which implies that *P* is commutative with *H*, namely [*H, P*] = 0. Another substantial property of the appropriate density matrix is particle conservation, Tr(*P*) = *N*_*e*_/2.

When the electronic temperature is zero, *f*(ε_*i*_) = 1 and the density matrix of insulator can be written as

(12) $$\begin{array}{l} P_{\mu \nu }=\overset{N_{e}}{\underset{i}{\sum }}c_{\mu i}c_{\nu i}^{*} \end{array}$$

which must satisfy the so-called idempotency *PP* = *P* with an orthogonal basis.

The solution of eigenvalue problem is under three restricted conditions of the density matrix mentioned above, which is known as the purification method. The trace-preserving canonical purification scheme of PM (Palser and Manolopoulos, 1998), which imposes commutation relation and trace-preserving condition, works with a predefined occupation and does not need the input or adjustment of the chemical potential. Trace-conserving spectral projections are performed during each iteration, until the density matrix *P*_*n*_ converges to the correct one that satisfies the idempotency condition. This method is inefficient at low and high partial occupancies (Palser and Manolopoulos, 1998; Daniels and Scuseria, 1999). A subsequent strategy proposed by Niklasson named TC algorithm (Niklasson, 2002). Its second-order form is called the second-order trace-correcting purification (TC2) method (Niklasson et al., 2003). The higher order TC2 requires additional matrix multiplications, which pursues a more rapid reduction of errors and a less step of purification iterations (Kim and Jung, 2016).

The TC2 purification algorithm is simple, robust, and rapidly convergent for closed-shell systems, and more efficient in orthogonal basis sets (Xiang et al., 2005). In this work, *H* denotes the Hamiltonian matrix under the presentation of orthogonal basis sets. Reasonably in the preparatory step, a transformation $H=Z^{\text{T}}H_{\text{AO}}Z$ is required, here the matrix *Z* is obtained by solving out the inverse square root of overlap matrix *S* by the Cholesky factorization (Cholesky, 2005). The idempotency and commutativity are satisfied naturally since the initial guess *P*_0_ is obtained by the Lanczos method (Lanczos, 1950).

**Figure 1:**
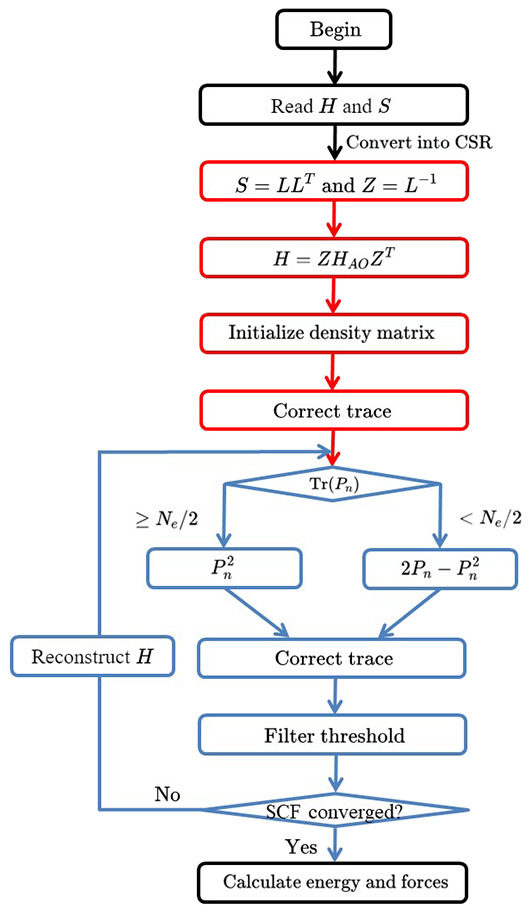
The flowchart of density matrix purification TC2 method. There are four time-consuming parts in the TC2 method, including constructing the Hamiltonian matrix, initializing the density matrix *P* from the Hamiltonian matrix with Cholesky and Lanzos methods, updating the density matrix with parallel sparse matrix–matrix multiplication, and computing total energy and atomic forces after SCF iterations.

During each iteration step, the trace of *P*_*n*+1_ is corrected by $P_{n}^{2}$ (Tr(*P*_*n*_) ≤ *N*_*e*_/2) or $2P_{n}-P_{n}^{2}$ (Tr(*P*_*n*_) > *N*_*e*_/2). Then, matrix elements less than a numerical threshold δ_filter_ (10^−4^ or 10^−6^) are dropped to zero, thus maintaining the sparsity of density matrix. The pseudocode of the TC2 algorithm is given in Algorithm 1 and its corresponding owchart is shown in Figure 1.

**Algorithm 1:** The pseudocode of TC2 algorithm, where *N*_*e*_ is the number of electrons, *E* is the energy-density matrix, ε_min_(*H*) and ε_max_(*H*) denote the minimal and maximum eigenvalue of the Hamiltonian matrix *H*, respectively.

**subroutine** TC2 (*H*, *P*, *N*_*e*_)
1: *S* = *LL*^*T*^
2: *Z* = *L*^−1^
3: $H=ZH_{AO}Z^{T}$
4: *P*_0_ = (ε_max_*I* − *H*)/(ε_max_ − ε_min_)
5: **do** iter = 1, niter
6: **if** Tr(*P*_*n*_) ≤ *N*_*e*_/2** then**
7: $P_{n+1}=P_{n}^{2}$
8: **else**
9: $P_{n+1}=2P_{n}-P_{n}^{2}$
10: δ = (Tr(*P*_*n*+1_*H*) − Tr(*P*_*n*_*H*))/Tr(*P*_*n*_*H*)
11: **enddo**
12: **if** (Converged) **then**
13: $P_{AO}=Z^{T}PZ$
14: $E=P_{AO}H_{AO}S^{-1}=P_{AO}H_{AO}Z^{T}Z$
15: **endif**
**end subrouine**

**Figure 2:**
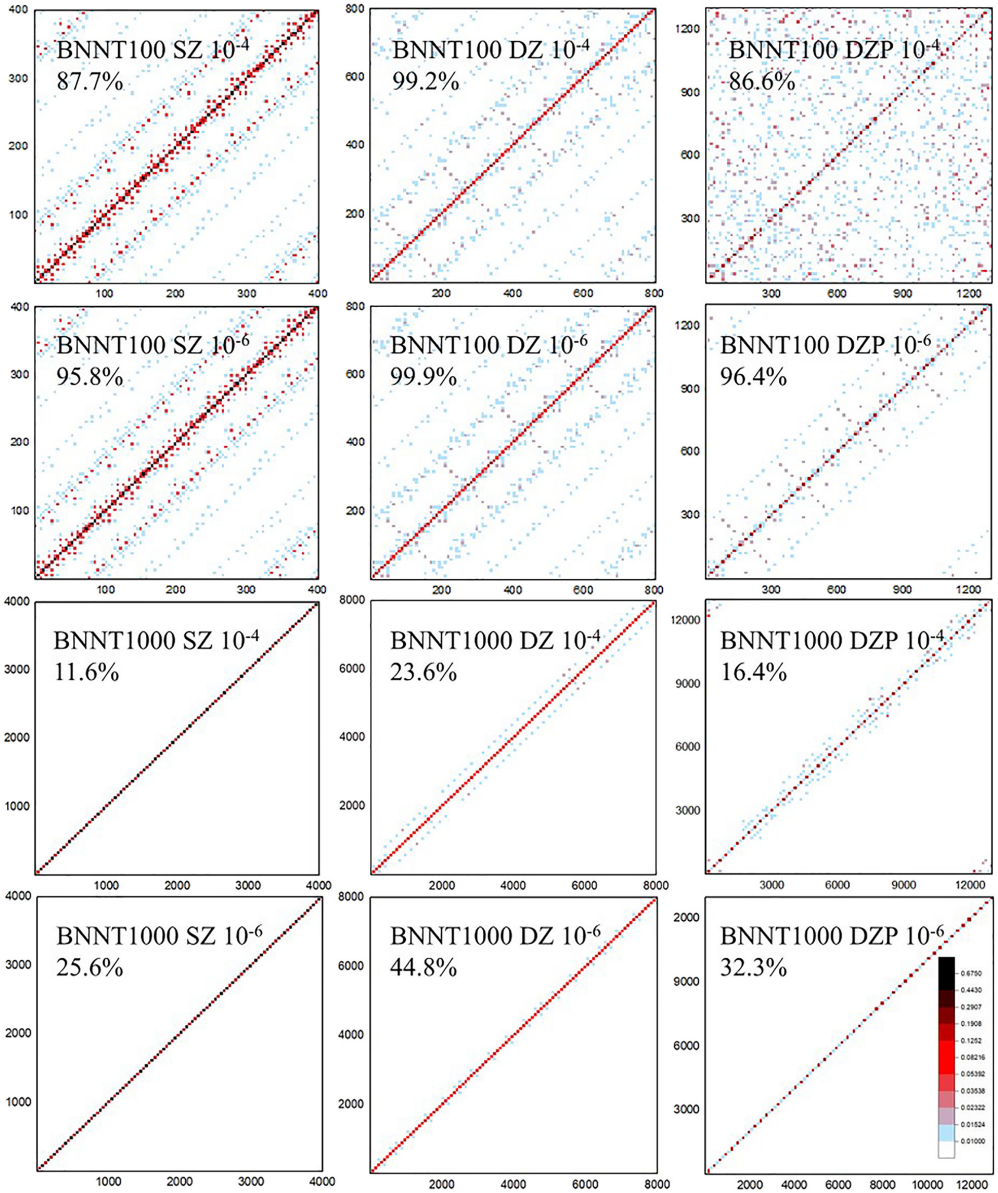
The sparsity of the density matrix *P* in the compressed sparse row (CSR) format of BN nanotubes (BNNTs) consisting of 100 and 1,000 atoms (BNNT100 and BNNT1000). Percentage under the system name indicates sparse degree of *P*. The matrix elements exceeding 10^−2^ are labeled as light blue and those exceeding 10^−1^ are marked as red. White area is remained for elements with tiny values or zero. Density matrix becomes less occupied with its elements gathering close to diagonal when the threshold after multiplication 100× tighter (10^−6^) than that of higher occupied matrix (10^−4^). When the basis sets become larger from SZ to DZP, sparse degree varies and δ_filter_ strongly affects the sparsity pattern.

The density matrix purification method in HONPAS is based on the fact that both the density matrix and Hamiltonian matrix are sparse with NAOs. Therefore, sparse matrix multiplication is the most expensive step in the density matrix purification method. Figure 2 shows the sparsity of the density matrix *P* saved as CSR format for the BN nanotubes consisting of 100 and 1,000 atoms (BNNT100 and BNNT1000) with different basis sets [single-ζ (SZ), double-ζ (DZ), and double-ζ plus polarization (DZP)] and thresholds (δ_filter_ = 10^−4^ and 10^−6^) of non-zero elements in the density matrix *P*.

When the selected basis sets are strictly localized, the coefficient matrix *C* for BNNT100 and BNNT1000 systems formed in a similar block diagonal matrix and arbitrary row of the resultant *P* is occupied by same number of non-zero elements since *P* = *CC*^*T*^. Therefore, the total number of non-zero elements grows linearly with the system size under tight binding approximation, which is the substantial precondition of almost all linear scaling algorithms. If we have an observation onto the first two columns of Figure 2, matrices show block-multidiagonal patterns and sparse degree of BNNT 1000 with the SZ basis set under δ_filter_ = 10^−4^ is 11.6%, which is obviously less than that of BNNT100 (87.7%). Variation trend of sparse degree is consistent with the character mentioned above from a perspective of image. Moreover, the cutoff radius *r*_*c*_ of the DZP basis set is higher, so non-zero elements per row distribute more intensively but their value is smaller than that of SZ and DZ. Sparse degree of BNNT1000 also decreases significantly from that of BNNT100 in the case of DZP basis set, such as 86.6% of BNNT100 and 16.4% of BNNT1000 at the same threshold, respectively.

On the perspective of threshold as shown in Figure 2, we observe that, as the system size increases, the influence of δ_filter_ is more significant, either the patterns or the sparse degree display an obvious difference under δ_filter_ = 10^−4^ and 10^−6^. For example, the density matrix of BNNT1000 with the DZP basis set is 32.3% occupied under δ_filter_ = 10^−6^ and 16.4% occupied under δ_filter_ = 10^−4^. On the other hand, δ_filter_ has a slight influence on the sparsity of BNNT100 compared with BNNT1000, which implies that the distribution of numerical value of matrix elements is shifted to higher level than that of BNNT1000. Just as elements dotted with deep color in BNNT100 are much more intensive than those of BNNT1000. It should be noted that dropping matrix elements less than δ_filter_ and using strictly truncated NAOs both contribute to the sparsity of *P*.

### 2.3. Parallel Implementation of Sparse Matrix Multiplication

The time required to process matrix–matrix multiplications during each iteration step accounts for a major part of total time. Note that there are some additional steps such as data communication and matrix addition. Fortunately, all matrices we need to deal with are sparse, so that the number of dot products reduces. The linear scaling cost arises from the fact that all matrix operations are performed on sparse matrices, which has a number of non-zero entries that increase linearly with the system size (VandeVondele et al., 2012).

The sparsity of matrix also causes unexpected drawbacks. An apparent disadvantage is, the matrix multiplication step would change the sparsity pattern during the self-consistent field (SCF) iterations, resulting in a load imbalance between matrix computation and data commutation among different processing cores. Since each matrix is distributed on a series of processes in advance, the instability of sparsity pattern will occur at each iteration, thus we also need to modify the data distribution after each iteration or exploit a block-cyclic distribution scheme. Apart from those, dropping matrix elements with the numerical value less than a threshold can reduce the number of dot products. But the computational accuracy of total energy and atomic forces is sacrificed inevitably under a loose threshold. The parallel version of TC2 algorithm in HONPAS is based on CSR data format and message-passing interface (MPI), which is capable of performing massive parallelism on modern heterogeneous supercomputers. We employ the SPARSEKIT library to manipulate and deal with sparse matrices, which provides programs for converting data structures, filtering out elements, and performing basic linear algebra operations with sparse matrix (Saad, 1994).

**Figure 3:**
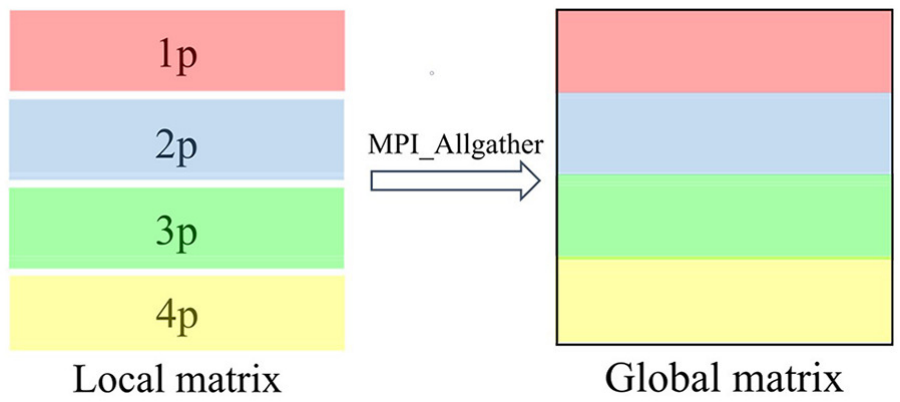
Parallel data distribution and communication of sparse matrix multiplication when *N*_*p*_ = 4. The density matrix is partitioned into four row block local matrices with 1D row BN nanotube (BNNT) grid parallelism (1p, 2p, 3p, and 4p). Each local matrix is stored in the compressed sparse row (CSR) format. MPI_Allgather is invoked to integrate these four row block local matrices into a global matrix in the CSR format.

In the parallel TC2 module, there is a single hierarchical structure of parallelization that consists of single type of data distribution and communication scheme. The TC2 module utilizes the MPI parallel programming to deal with data communications between different MPI processes. In our work, the MPI processes are organized in 1D row MPI grids. The density matrix is distributed by 1D row blocks across MPI processes, and each process saves *N*_b_/*N*_p_ rows of global matrix. Thus, such local and global sparse matrix–matrix multiplication does not require additional data communication. Individual process computes its part of the multiplication, processing a row block of np (*n* = 1, 2, …, *N*_p_) at a time. After the local multiplication has been processed, each processor just gathers a local subset of global density matrix. We use the MPI_Allgather function to gather local matrices into global density matrix in each MPI process, similar to the case of MPI_Gather and then MPI_Bcast, then performing local sparse matrix–matrix multiplication at the next iteration step. Figure 3 illustrates the schematic diagram of MPI communication on CSR data format, in which we set *N*_p_ = 4 to simplify the discussion.

## 3. Results and Discussion

In this section, we demonstrate the computational accuracy and efficiency of our parallel TC2 algorithm. We implement this method in the HONPAS package (Qin et al., 2015), which has been written in the Fortran programming language with the MPI for parallelism. We use the norm-conserving Troullier-Martins pseudopotentials (Troullier and Martins, 1991) to represent interaction between core and valence electrons. We use the exchange-correlation functional of local density approximation of Goedecker-Teter-Hutter (LDA-PZ) (Goedecker et al., 1996) to describe the electronic structures of these BNNTs with a grid cutoff of 100 Ry. In our calculations, the NAOs are generated by default parameters in SIESTA. We utilize the linear-scaling density matrix TC2 purification algorithm to calculate the electronic structures of a series of boron nitride nanotubes (BNNTs), containing 100–18,000 atoms (labeled by BNNT100-BNNT18000). The details of the input parameters and atomic structures of BNNTs used in this work as well as the performance data are shown in the Supplementary Materials.

### 3.1. Accuracy

We benchmark the computational accuracy of parallel TC2 method by comparing the results with those obtained from the diagnonalization method. We consider the effects of both the size of basis sets (SZ, DZ, and DZP) and different values of thresholds (δ_filter_ = 10^−4^ to 10^−6^) on the computational accuracy of density matrix TC2 purification algorithm. We define the errors of total energy and atomic forces, respectively, as

$$\begin{array}{l} \Delta E_{\text{tot}}=|\Delta E^{\text{TC2}}-E^{\text{DIAG}}|/N_{\text{A}}\\ \;\Delta F_{I}=|F_{I}^{\text{TC2}}-F_{I}^{\text{DIAG}}| \end{array}$$

where *N*_*A*_ is the total number of atoms and *I* is the atom index.

**Figure 4:**
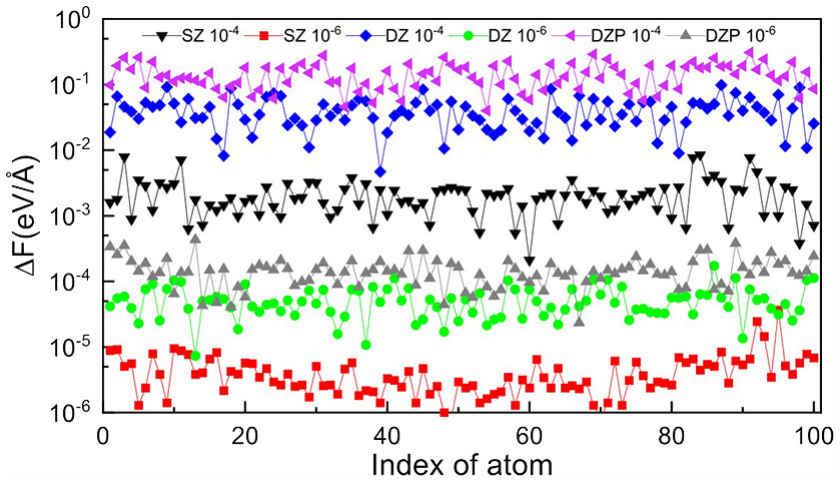
Variation of root mean square error of atomic force on each atom computed with the TC2 and diagonalization methods with different basis sets (SZ, DZ, and DZP) and thresholds (δ_filter_ = 10^−4^ and 10^−6^) for BNNT100.

In the HONPAS calculations, the default convergence accuracy for total energy and atomic forces are 10^−4^ eV/atom and 0.02 eV/Å, respectively. Table 1 shows that the TC2 purification calculation for total energy is performed very well when choosing a tight dropping threshold, and $\delta_{\text{filter}}=10^{-6}$ can yield a total energy accuracy of 10^−5^ eV/atom at least. On the other hand, strictly truncated NAOs can yield the sparsity without loss of accuracy simultaneously (Shang et al., 2010). We compute the total energy and atomic forces under different basis sets using a variable threshold. As shown in Figure 4, the errors of atomic forces from TC2 and those obtained from direct diagonalization method are indistinguishable. For all tested systems, the accuracy of the TC2 method can be obviously improved by tightening the threshold (10^−4^ to 10^−6^). In particular, when the threshold is set to 10^−4^, Δ*F*_max_ with the most general case of DZP basis set reaches 10^−1^ eV/Å, which is already comparable to the magnitude of atomic force itself. In contrast, $\Delta F_{\text{max}}=4.30\times 10^{-4}$ eV/Å under $\delta_{\text{filter}}=10^{-6}$ with the same basis set. The noticeable error arises from the lack of information in density matrix when too many elements are neglected after each iteration step and the information of Hamiltonian matrix just included in the initial step. However, the relative error of energy per atom is less than 10^−7^ when threshold is set to 10^−6^ in the case of SZ basis set, which indicates that the computational accuracy of TC2 method is still guaranteed. On the perspective of basis sets, high accuracy is ensured when we employ rigorously localized basis sets (SZ). Note that systems with the DZP basis set have relatively larger errors, since the information of polarization orbital is partly omitted by dropping matrix elements. For instance, when δ_filter_ is set to 10^−4^, the energy error for SZ is 3.36 × 10^−4^ but that for DZP is 8.80 × 10^−3^ (still can achieve the converged accuracy). As we have mentioned in section 2, non-zero elements those hold relatively small value distribute more intensively in the case of DZP basis set, and the physical information can be seriously lacking under relatively large δ_filter_.

**Table 1:** Absolute error of total energy Δ*E*_tot_ (eV/atom) and the maximum of root mean square error of atomic forces Δ*F*_max_ (eV/Å) of the TC2 method with varying thresholds of δ_filter_ = 10^−4^ and 10^−6^ for the BNNT100 system with SZ, DZ, and DZP basis sets.

**Basis sets**	**δ_filter_**	**Δ*E*_tot_**	**Δ*F*_max_**
SZ	10^−4^	3.36 × 10^−4^	8.48 × 10^−3^
DZ	10^−4^	2.26 × 10^−3^	9.80 × 10^−2^
DZP	10^−4^	8.80 × 10^−3^	3.07 × 10^−1^
SZ	10^−6^	3.70 × 10^−7^	3.64 × 10^−5^
DZ	10^−6^	4.72 × 10^−5^	1.73 × 10^−4^
DZP	10^−6^	1.06 × 10^−5^	4.30 × 10^−4^

*The reference results are computed by the direct diagonalization method*.

### 3.2. Efficiency

**Figure 5:**
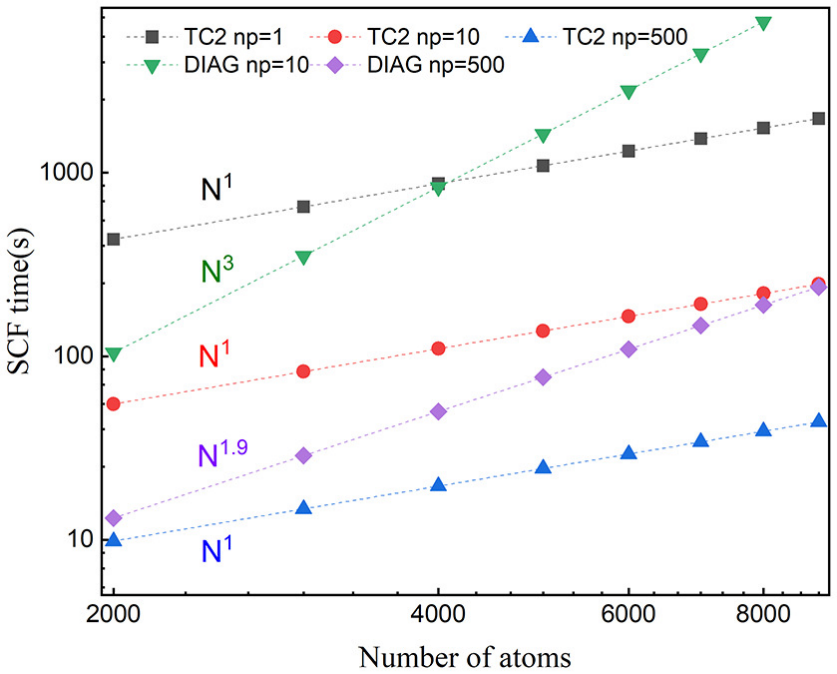
Weak scaling of wall clock time per SCF iteration with respect to the number of atoms with the message-passing interface (MPI) parallelism for BN nanotubes (BNNTs) with 2,000 and 9,000 atoms (BNNT2000 and BNNT9000) computed with the TC2 and diagonalization methods.

**Figure 6:**
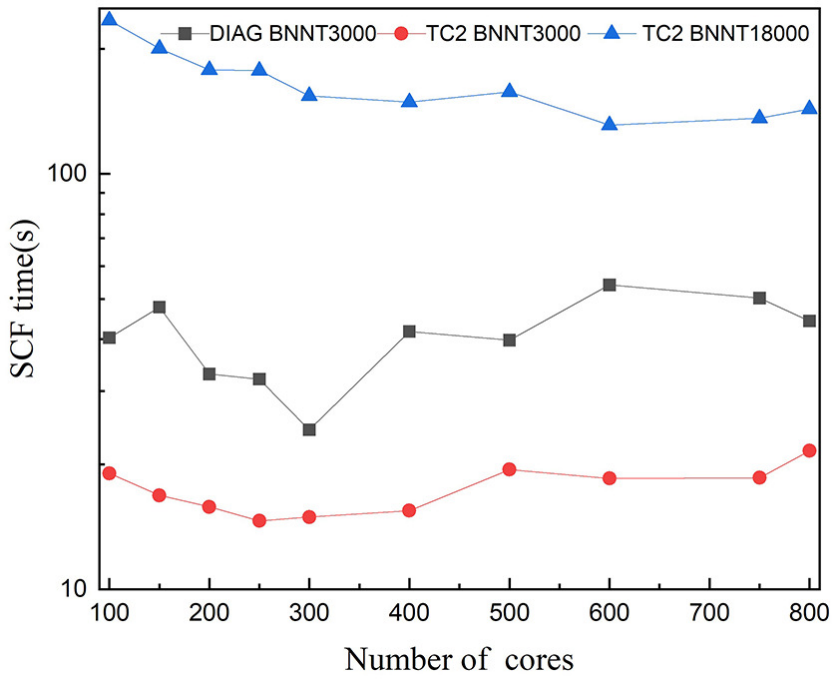
Strong scaling of wall clock time per SCF iteration with respect to the number of cores with the message-passing interface (MPI) parallelism for BN nanotubes (BNNTs) with 3,000 and 18,000 atoms (BNNT3000 and BNNT18000) computed with the TC2 and diagonalization methods.

We demonstrate the computational efficiency and parallel scalability of linear-scaling TC2 method by checking the weak and strong scaling performance on BNNT systems with the SZ basis set and a threshold of $\delta_{\text{filter}}=10^{-4}$. We illustrate the total time of the main time-consuming parts as shown in Figure 1: (a) Construction of Hamiltonian matrix, (b) evaluation of density matrix *P* from Hamiltonian matrix by Cholesky factorization following Lanczos method, and (c) purification with matrix multiplication and addition. It should be noticed that the data communication via MPI interface also occupies numerous time resource while performing massive parallelization over plenty of processing cores. Practical tests on the computational efficiency and parallel scalability are performed in the case of BNNT systems with MPI parallelism on modern heterogeneous supercomputers, including comparison of TC2 and diagonalization methods with respect to different system sizes and process counts, as shown in Figures 5, 6, respectively. There are some additional steps such as computing total energy and atomic forces, which are all included in the total wall clock time of outer SCF iterations in the TC2 method.

Since the computational cost of linear-scaling TC2 method grows linearly with respect to the system size, a noteworthy speed up is supposed to be observed. We choose all tested systems with the SZ basis set to illustrate strong and weak scaling behaviors, since it is more strictly localized, resulting in a relatively small change of sparsity pattern after each iteration step. The variation of total time with respect to the system size is plotted in Figure 5. We can see that the scaling of TC2 is fitted to *O*(*N*) due to the linearly growing sparse degree of *P*, and the number required to perform multiplication has the same trend. Linear scaling behavior is obtained with various systems containing 2,000–9,000 atoms under serial mode (*N*_*p*_ = 1), and it continues to scale further up to 500 cores at least, which benefits from the efficient parallel implementation of matrix multiplications based on the CSR formatted sparse density matrix. A speed-up of 4.7 can be achieved for 9,000 atoms (500 cores) and could be larger for more atoms. The fitted scaling for explicit diagonalization is just *O*(*N*^1.9^) with number of atoms fewer than 5,000 when the number of processors is relatively large, arising from the load imbalance that problem size (number of computational tasks) distributed on each process is not adequate and some cores remain idle. If the processors keep increasing, low efficiency of parallelization is going to happen. When the size of system grows sufficiently or processing cores have a relevant scale, fitted scaling turns back to *O*(*N*^3^) due to the cubic scaling of conventional diagonalization step. As a conclusion, linear-scaling TC2 method outperforms explicit diagonalization in terms of expansibility to large systems and massive parallel implementation.

Figure 6 compares the parallel scalability of TC2 to diagonalization methods. As it can be seen, the parallel scalability of both methods is unsatisfactory, especially with the smaller system size. This issue arises in the load imbalance caused by idle processors since computational tasks are inadequate compared with hundreds of cores. Test for 18,000 atoms with diagonalization is not represented due to a memory overflow problem (the dimension of matrix is 72,000). Unlike the diagonalization method, test for TC2 has been performed since the utilization of CSR data format reduces the memory requirement. TC2 method demonstrates just scaling up to 600 cores, since the 1D processes layout prevents it from massive parallelization. The performance of global MPI communications such as MPI_Allgather is strongly impacted by the physical distance of remote processing cores, which prompts us to utilize BCSR storage format and 2D block-cyclic processor layout.

## 4. Conclusion and Outlook

In summary, we present a parallel implementation of linear-scaling density matrix trace correcting (TC) purification algorithm to solve KS equations with numerical atomic orbitals in the HONPAS package. We use the MPI_Allgather function for parallel programming to deal with the sparse matrix multiplication within the CSR format, which can scale up to hundreds of processing cores on modern heterogeneous supercomputers. We demonstrate the computational accuracy and efficiency of this linear-scaling density matrix purification algorithm by performing large-scale DFT calculations on boron nitrogen nanotubes containing tens of thousands of atoms.

However, our parallel implementation of TC2 method in HONPAS is inferior to that of BigDFT (Genovese et al., 2008; Mohr et al., 2014), ONETEP (Skylaris et al., 2005), and CONQUEST (Gillan et al., 2007). They exploit more than one level of organization and data distribution schemes resembling the BCSR format to handle the groups of atoms, which achieve high flexibility in load balancing (Bowler et al., 2002) with high performance on modern heterogeneous supercomputers. In the future, We plan to implement a massively parallel algorithm based on the NTPoly library (Dawson and Nakajima, 2018) in HONPAS. The NTPoly library utilizes the 3D sparse matrix multiplication algorithm, that is, the processors are organized into a three dimensional, cube-shaped virtual topology. In this case, density matrix purification algorithms can scale up to thousands of processing cores on modern heterogeneous supercomputers.

## Data Availability Statement

All datasets presented in this study are included in the article/Supplementary Material.

## Author Contributions

ZL has tested the computational accuracy and efficiency of the TC2 method in HONPAS package, written manuscript and drawn figures. WH and XQ provide the research direction and revise the manuscript. WH has provided codes of the parallel version of TC2 method in HONPAS and directive methods for tests of computational accuracy and efficiency. LW has offered help for the modification of formulas. All authors contributed to the article and approved the submitted version.

## Conflict of Interest

The authors declare that the research was conducted in the absence of any commercial or financial relationships that could be construed as a potential conflict of interest.
