# Supplementary material for: Parallel Implementation of Large-Scale Linear Scaling Density Functional Theory Calculations With Numerical Atomic Orbitals in HONPAS
Source: Front Chem. 2020 Nov 26;8:589910. doi: 10.3389/fchem.2020.589910 (PMC7726133; doi:10.3389/fchem.2020.589910)
Supplement: Supplementary file 1 [file Data_Sheet_1.PDF]

Input file

The file of coordinate: coord.fdf

```
NumberOfAtoms      100
LatticeConstant             1 Ang
%block LatticeParameters
10.2702     10.2702     12.5574     90.0000     90.0000    120.0000
%endblock LatticeParameters
AtomicCoordinatesFormat    NotScaledCartesianAng
%block AtomicCoordinatesAndAtomicSpecies
  5.512501618     6.266521202     1.077039399   2   N    1
  1.747266831     7.810168649     1.077039399   2   N    2
  4.517890818     7.307043477     1.077039399   1   B    3
  3.880664954     7.650044397     2.332776235   2   N    4
  0.450319702     7.185776632     1.077039399   1   B    5
-0.072807180     6.685731935     2.332776235   2   N    6
  2.464404267     7.907227040     2.332776235   1   B    7
-0.884350668     4.706230864     1.077039399   2   N    8
-0.755051030     5.418261787     2.332776235   1   B    9
  1.254455058     1.244244367     1.077039399   2   N   10
-0.691297277     3.279813101     1.077039399   1   B   11
-0.377381606     2.627767562     2.332776235   2   N   12
  2.670715745     0.987061724     1.077039399   1   B   13
  3.387853181     1.084120116     2.332776235   2   N   14
  0.617229194     1.587245287     2.332776235   1   B   15
  5.207927192     2.208556829     1.077039399   2   N   16
  4.684800311     1.708512132     2.332776235   1   B   17
  5.890171042     3.476026977     1.077039399   1   B   18
  6.019470681     4.188057900     2.332776235   2   N   19
  5.826417289     5.614475663     2.332776235   1   B   20
  5.512501618     6.266521202     3.588513070   2   N   21
  1.747266831     7.810168649     3.588513070   2   N   22
  4.517890818     7.307043477     3.588513070   1   B   23
  3.880664954     7.650044397     4.844249906   2   N   24
  0.450319702     7.185776632     3.588513070   1   B   25
-0.072807180     6.685731935     4.844249906   2   N   26
  2.464404267     7.907227040     4.844249906   1   B   27
-0.884350668     4.706230864     3.588513070   2   N   28
-0.755051030     5.418261787     4.844249906   1   B   29
  1.254455058     1.244244367     3.588513070   2   N   30
-0.691297277     3.279813101     3.588513070   1   B   31
-0.377381606     2.627767562     4.844249906   2   N   32
```

2.670715745	0.987061724	3.588513070	1	B	33
3.387853181	1.084120116	4.844249906	2	N	34
0.617229194	1.587245287	4.844249906	1	B	35
5.207927192	2.208556829	3.588513070	2	N	36
4.684800311	1.708512132	4.844249906	1	B	37
5.890171042	3.476026977	3.588513070	1	B	38
6.019470681	4.188057900	4.844249906	2	N	39
5.826417289	5.614475663	4.844249906	1	B	40
5.512501618	6.266521202	6.099986741	2	N	41
1.747266831	7.810168649	6.099986741	2	N	42
4.517890818	7.307043477	6.099986741	1	B	43
3.880664954	7.650044397	7.355723577	2	N	44
0.450319702	7.185776632	6.099986741	1	B	45
-0.072807180	6.685731935	7.355723577	2	N	46
2.464404267	7.907227040	7.355723577	1	B	47
-0.884350668	4.706230864	6.099986741	2	N	48
-0.755051030	5.418261787	7.355723577	1	B	49
1.254455058	1.244244367	6.099986741	2	N	50
-0.691297277	3.279813101	6.099986741	1	B	51
-0.377381606	2.627767562	7.355723577	2	N	52
2.670715745	0.987061724	6.099986741	1	B	53
3.387853181	1.084120116	7.355723577	2	N	54
0.617229194	1.587245287	7.355723577	1	B	55
5.207927192	2.208556829	6.099986741	2	N	56
4.684800311	1.708512132	7.355723577	1	B	57
5.890171042	3.476026977	6.099986741	1	B	58
6.019470681	4.188057900	7.355723577	2	N	59
5.826417289	5.614475663	7.355723577	1	B	60
5.512501618	6.266521202	8.611460412	2	N	61
1.747266831	7.810168649	8.611460412	2	N	62
4.517890818	7.307043477	8.611460412	1	B	63
3.880664954	7.650044397	9.867197248	2	N	64
0.450319702	7.185776632	8.611460412	1	B	65
-0.072807180	6.685731935	9.867197248	2	N	66
2.464404267	7.907227040	9.867197248	1	B	67
-0.884350668	4.706230864	8.611460412	2	N	68
-0.755051030	5.418261787	9.867197248	1	B	69
1.254455058	1.244244367	8.611460412	2	N	70
-0.691297277	3.279813101	8.611460412	1	B	71
-0.377381606	2.627767562	9.867197248	2	N	72
2.670715745	0.987061724	8.611460412	1	B	73
3.387853181	1.084120116	9.867197248	2	N	74
0.617229194	1.587245287	9.867197248	1	B	75
5.207927192	2.208556829	8.611460412	2	N	76

```
  4.684800311       1.708512132       9.867197248   1   B   77
  5.890171042       3.476026977       8.611460412   1   B   78
  6.019470681       4.188057900       9.867197248   2   N   79
  5.826417289       5.614475663       9.867197248   1   B   80
  5.512501618       6.266521202      11.122934083   2   N   81
  1.747266831       7.810168649      11.122934083   2   N   82
  4.517890818       7.307043477      11.122934083   1   B   83
  3.880664954       7.650044397      12.378670919   2   N   84
  0.450319702       7.185776632      11.122934083   1   B   85
 -0.072807180       6.685731935      12.378670919   2   N   86
  2.464404267       7.907227040      12.378670919   1   B   87
 -0.884350668       4.706230864      11.122934083   2   N   88
 -0.755051030       5.418261787      12.378670919   1   B   89
  1.254455058       1.244244367      11.122934083   2   N   90
 -0.691297277       3.279813101      11.122934083   1   B   91
 -0.377381606       2.627767562      12.378670919   2   N   92
  2.670715745       0.987061724      11.122934083   1   B   93
  3.387853181       1.084120116      12.378670919   2   N   94
  0.617229194       1.587245287      12.378670919   1   B   95
  5.207927192       2.208556829      11.122934083   2   N   96
  4.684800311       1.708512132      12.378670919   1   B   97
  5.890171042       3.476026977      11.122934083   1   B   98
  6.019470681       4.188057900      12.378670919   2   N   99
  5.826417289       5.614475663      12.378670919   1   B   100
%endblock AtomicCoordinatesAndAtomicSpecies
```

The standard input file: BN.fdf

```
SystemName          BN(5,5)
SystemLabel         BN(5,5)
NumberOfSpecies     2

%block ChemicalSpeciesLabel
  1   5   B         # Species index, atomic number, species label
  2   7   N
%endblock ChemicalSpeciesLabel

%block PS.lmax
B 1
N 1
%endblock PS.lmax

%include coord.fdf
#PAO.BasisSize DZP

PAO.BasisSize SZ
```

```
SolutionMethod      dm_on
%include DM.fdf
#%include DMPT.fdf
#SolutionMethod      diagon
MeshCutoff                   100.0000000          Ry
#MD.TypeOfRun FC
#MD.NumCGsteps 50
#WriteForces
#MD.MaxForceTol 0.01 eV/Ang
#DM.UseSaveDM
DM.MixingWeight 0.4
DM.NumberPulay 6
```

Input file acquired for TC2: DM.fdf

```
#linear scaling DM
DM_method 5
maxtrix_filter .TRUE.
maxtrix_filter_tolerance 1.0d-4
#for spin polarized calculation
#TRS4_DM_SP    .TRUE.

#USE_BCSR
M_divide_basis 4
na_block 10
AINV_tolerance    1.0d-10
#USE_Block_Ainv
DM_max_iteration    500
MaxSCFIterations 2
use_nofix_sym_mul .FALSE.
#use_nofix_sym_mul
#reorder_atoms

#DM_fix_SF    .TRUE.
DM_fix_SF    .FALSE.
#DM_cutoff    2.2
USE_fix_sym_mul .TRUE.
Neg_Blk_Mul    0.1
TRS4_ONLY_TC2 .TRUE.
#DM_saveall
#DM_writeall
#DM_readall

#linear scaling DMPT
```

Parallel efficiency

N_p	DIAG BNNT3000	TC2 BNNT3000	TC2 BNNT18000
100	40.326	19.016	234.349
150	47.756	16.833	200.407
200	32.976	15.797	177.983
250	32.05	14.615	177.182
300	24.212	14.935	153.929
400	41.747	15.468	148.709
500	39.769	19.423	157.352
600	54.034	18.493	130.906
750	50.241	18.565	136.033
800	44.244	21.558	143.171

Table S1 Strong scaling of wall clock time per SCF iteration with respect to the number of cores with the MPI parallelism for BNNTs with 3000 and and 18000 atoms (BNNT3000 and BNNT18000) computed with the TC2 and diagonalization methods.The unit of time is s.

atoms	TC2 np=1	TC2 np=10	TC2 np=500	DIAG np=500	DIAG np=10
2000	434.447	54.496	9.773	14.83	102.831
3000	651.542	81.551	14.655	28.96	344.359
4000	873.24	109.926	19.93	43.396	818.079
5000	1087.551	144.912	24.559	65.074	1905.149
6000	1306.035	163.105	30.213	104.749	2655.959
7000	1524.802	190.914	33.074	172.631	4414.477
8000	1770.852	218.611	38.778	188.616	6371.389
9000	1966.821	244.294	44.013	255.069	

Table S2 Weak scaling of wall clock time per SCF iteration with respect to the number of atoms with the MPI parallelism for BNNTs with 2000 and 9000 atoms (BNNT2000 and BNNT9000) computed with the TC2 and diagonalization methods. The unit of time is s.

Accuracy

SZ basis set

$\delta_{\text{filter}} = 10^{-4}$: TC2

siesta: Final energy (eV):

siesta:	Kinetic =	12539.358164
siesta:	Hartree =	9464.161610
siesta:	Ext. field =	0.000000
siesta:	Exch.-corr. =	-5191.604129
siesta:	Ion-electron =	-30280.469709
siesta:	Ion-ion =	-4091.947116
siesta:	Ekinion =	0.000000
siesta:	Total =	-17560.501180

siesta: Atomic forces (eV/Ang):

siesta:	1	0.718594	0.189116	-0.786225
siesta:	2	-0.137498	0.810630	0.049918
siesta:	3	-0.161255	0.144725	0.023426
siesta:	4	0.702796	0.325988	0.176129
siesta:	5	-0.266480	-0.253212	0.001330
siesta:	6	-0.174668	0.092700	-0.031095
siesta:	7	-0.329714	-0.120601	-0.043766
siesta:	8	-0.798099	-0.034612	0.036887
siesta:	9	0.029613	-0.369731	0.014225
siesta:	10	-0.698660	-0.323442	0.171974
siesta:	11	0.195709	-0.235203	0.009565
siesta:	12	-0.720288	-0.188606	-0.784658
siesta:	13	0.331193	0.122675	-0.040987
siesta:	14	0.137338	-0.810715	0.053649
siesta:	15	0.165541	-0.152661	0.013569
siesta:	16	0.177330	-0.095946	-0.035229
siesta:	17	0.264803	0.251681	0.009050
siesta:	18	-0.032201	0.370145	0.013285
siesta:	19	0.800862	0.034728	0.038439
siesta:	20	-0.199138	0.241684	0.004615
siesta:	21	0.719517	0.191052	-0.785508
siesta:	22	-0.137113	0.810168	0.055426
siesta:	23	-0.168793	0.146685	0.012525
siesta:	24	0.702400	0.327530	0.176458
siesta:	25	-0.262732	-0.250373	0.006554
siesta:	26	-0.178354	0.092172	-0.033643

siesta:	27	-0.333105	-0.122962	-0.045500
siesta:	28	-0.801830	-0.038375	0.041058
siesta:	29	0.033645	-0.362983	0.015460
siesta:	30	-0.700653	-0.324388	0.179952
siesta:	31	0.200875	-0.244169	0.001073
siesta:	32	-0.718937	-0.186900	-0.783194
siesta:	33	0.332953	0.122749	-0.042159
siesta:	34	0.138389	-0.813924	0.050655
siesta:	35	0.166057	-0.150867	0.007555
siesta:	36	0.178998	-0.096270	-0.032305
siesta:	37	0.260065	0.252955	0.008750
siesta:	38	-0.032496	0.367219	0.011042
siesta:	39	0.796882	0.035595	0.042025
siesta:	40	-0.199336	0.244808	0.005185
siesta:	41	0.723080	0.191955	-0.784027
siesta:	42	-0.136688	0.809372	0.055984
siesta:	43	-0.168099	0.148701	0.012303
siesta:	44	0.702792	0.326335	0.176902
siesta:	45	-0.262780	-0.248212	0.008226
siesta:	46	-0.176105	0.096245	-0.034339
siesta:	47	-0.332762	-0.123367	-0.046796
siesta:	48	-0.801864	-0.039146	0.043951
siesta:	49	0.032961	-0.362969	0.010584
siesta:	50	-0.701707	-0.324408	0.178788
siesta:	51	0.198680	-0.241834	0.000098
siesta:	52	-0.719646	-0.189312	-0.782315
siesta:	53	0.331949	0.122510	-0.043621
siesta:	54	0.139483	-0.814406	0.052842
siesta:	55	0.166867	-0.151397	0.010504
siesta:	56	0.179785	-0.097232	-0.034749
siesta:	57	0.260564	0.252119	0.008695
siesta:	58	-0.033733	0.367597	0.011594
siesta:	59	0.800002	0.033620	0.040438
siesta:	60	-0.199336	0.244348	0.003542
siesta:	61	0.723048	0.189124	-0.781954
siesta:	62	-0.137828	0.808761	0.056541
siesta:	63	-0.168110	0.147704	0.012279
siesta:	64	0.701235	0.325515	0.173693
siesta:	65	-0.261771	-0.250880	0.009536
siesta:	66	-0.181213	0.095516	-0.036324
siesta:	67	-0.330894	-0.121727	-0.046842
siesta:	68	-0.802607	-0.037502	0.041065
siesta:	69	0.034932	-0.363793	0.012893
siesta:	70	-0.702234	-0.324185	0.177426

```
siesta:     71    0.200815   -0.242793    0.002780
siesta:     72   -0.718897   -0.189721   -0.783980
siesta:     73    0.328960    0.125091   -0.043501
siesta:     74    0.139383   -0.809716    0.055985
siesta:     75    0.169204   -0.150668    0.016553
siesta:     76    0.179736   -0.095506   -0.034632
siesta:     77    0.260356    0.251363    0.007290
siesta:     78   -0.033888    0.365491    0.011559
siesta:     79    0.796538    0.034024    0.042410
siesta:     80   -0.198242    0.243536    0.003544
siesta:     81    0.724416    0.188499   -0.786114
siesta:     82   -0.138173    0.810160    0.053240
siesta:     83   -0.177234    0.155986    0.021597
siesta:     84    0.701740    0.326560    0.161033
siesta:     85   -0.260071   -0.245958    0.008195
siesta:     86   -0.176116    0.103111   -0.028285
siesta:     87   -0.331241   -0.123413   -0.036794
siesta:     88   -0.802470   -0.034963    0.041913
siesta:     89    0.035913   -0.368242    0.016203
siesta:     90   -0.704216   -0.329058    0.172227
siesta:     91    0.205081   -0.253450    0.011388
siesta:     92   -0.721115   -0.187662   -0.790976
siesta:     93    0.331204    0.122276   -0.041438
siesta:     94    0.137314   -0.813246    0.049211
siesta:     95    0.168515   -0.150067    0.013675
siesta:     96    0.181158   -0.098441   -0.032779
siesta:     97    0.262037    0.249265    0.011174
siesta:     98   -0.033034    0.366858    0.012644
siesta:     99    0.799031    0.036085    0.042304
siesta:    100   -0.200881    0.245116    0.004092
siesta: ----------------------------------------
siesta:    Tot    0.006034   -0.001366   -5.527056

siesta: Stress tensor (static) (eV/Ang**3):
siesta:     -0.036870    0.000302   -0.000007
siesta:      0.001276   -0.039704    0.000023
siesta:      0.000008    0.000014   -0.051252

siesta: Cell volume =       1147.065353 Ang**3

siesta: Pressure (static):
siesta:                Solid           Molecule  Units
siesta:           0.00046406          0.00016461  Ry/Bohr**3
siesta:           0.04260826          0.01511380  eV/Ang**3
```

siesta: 68.26670271 24.21524198 kBar

$\delta_{\text{filter}} = 10^{-4}$: DIAG

siesta: Final energy (eV):
siesta: Kinetic = 12539.102064
siesta: Hartree = 9463.951289
siesta: Ext. field = 0.000000
siesta: Exch.-corr. = -5191.559678
siesta: Ion-electron = -30280.081383
siesta: Ion-ion = -4091.947116
siesta: Ekinion = 0.000000
siesta: Total = -17560.534825

siesta: Atomic forces (eV/Ang):
siesta: 1 0.720448 0.188572 -0.784244
siesta: 2 -0.137351 0.810833 0.052976
siesta: 3 -0.167768 0.151177 0.013178
siesta: 4 0.701846 0.325743 0.174947
siesta: 5 -0.264486 -0.250425 0.006338
siesta: 6 -0.175971 0.096975 -0.033310
siesta: 7 -0.331165 -0.122021 -0.043242
siesta: 8 -0.801342 -0.035041 0.041284
siesta: 9 0.032998 -0.367385 0.011965
siesta: 10 -0.702457 -0.326364 0.174245
siesta: 11 0.199832 -0.244503 0.002713
siesta: 12 -0.720065 -0.188065 -0.783740
siesta: 13 0.331491 0.122142 -0.043946
siesta: 14 0.138541 -0.810928 0.053882
siesta: 15 0.167473 -0.151071 0.013905
siesta: 16 0.176294 -0.096961 -0.033724
siesta: 17 0.264330 0.250226 0.007048
siesta: 18 -0.032951 0.367726 0.011252
siesta: 19 0.801023 0.034296 0.040070
siesta: 20 -0.199659 0.244255 0.003440
siesta: 21 0.720335 0.188799 -0.783422
siesta: 22 -0.137617 0.810800 0.053821
siesta: 23 -0.167496 0.151046 0.013980
siesta: 24 0.701804 0.325752 0.174983
siesta: 25 -0.264279 -0.250202 0.007136
siesta: 26 -0.175968 0.097024 -0.033329
siesta: 27 -0.331175 -0.122045 -0.043190
siesta: 28 -0.801378 -0.035308 0.042084
siesta: 29 0.033021 -0.367398 0.012028
siesta: 30 -0.702155 -0.326528 0.175068

siesta:	31	0.199679	-0.244236	0.003518
siesta:	32	-0.720058	-0.187990	-0.783708
siesta:	33	0.331195	0.122082	-0.043152
siesta:	34	0.138513	-0.810908	0.053930
siesta:	35	0.167490	-0.151058	0.013966
siesta:	36	0.176529	-0.096809	-0.032970
siesta:	37	0.264320	0.250252	0.007109
siesta:	38	-0.032988	0.367422	0.012054
siesta:	39	0.801134	0.034934	0.042240
siesta:	40	-0.199684	0.244253	0.003503
siesta:	41	0.720381	0.188733	-0.783489
siesta:	42	-0.137582	0.810825	0.053774
siesta:	43	-0.167504	0.151054	0.013963
siesta:	44	0.701756	0.325785	0.174941
siesta:	45	-0.264295	-0.250210	0.007116
siesta:	46	-0.175992	0.097082	-0.033429
siesta:	47	-0.331169	-0.122046	-0.043206
siesta:	48	-0.801373	-0.035266	0.041997
siesta:	49	0.033022	-0.367395	0.012024
siesta:	50	-0.702128	-0.326568	0.175006
siesta:	51	0.199682	-0.244247	0.003497
siesta:	52	-0.720078	-0.187933	-0.783741
siesta:	53	0.331205	0.122081	-0.043187
siesta:	54	0.138487	-0.810911	0.053911
siesta:	55	0.167485	-0.151056	0.013962
siesta:	56	0.176535	-0.096895	-0.033079
siesta:	57	0.264319	0.250251	0.007104
siesta:	58	-0.033023	0.367391	0.012172
siesta:	59	0.801123	0.034899	0.042182
siesta:	60	-0.199685	0.244252	0.003498
siesta:	61	0.720397	0.188672	-0.783529
siesta:	62	-0.137564	0.810838	0.053754
siesta:	63	-0.167501	0.151053	0.013960
siesta:	64	0.701693	0.325815	0.174879
siesta:	65	-0.264299	-0.250212	0.007111
siesta:	66	-0.176008	0.097133	-0.033547
siesta:	67	-0.331149	-0.122046	-0.043234
siesta:	68	-0.801358	-0.035231	0.041935
siesta:	69	0.033024	-0.367386	0.012010
siesta:	70	-0.702088	-0.326600	0.174964
siesta:	71	0.199681	-0.244247	0.003492
siesta:	72	-0.720083	-0.187893	-0.783801
siesta:	73	0.331199	0.122079	-0.043202
siesta:	74	0.138478	-0.810911	0.053873

siesta: 75 0.167472 -0.151048 0.013947
siesta: 76 0.176678 -0.097010 -0.033318
siesta: 77 0.264313 0.250245 0.007085
siesta: 78 -0.033020 0.367392 0.012167
siesta: 79 0.801104 0.034895 0.042095
siesta: 80 -0.199678 0.244235 0.003481
siesta: 81 0.720449 0.188641 -0.783483
siesta: 82 -0.137541 0.810887 0.053815
siesta: 83 -0.167477 0.151060 0.014022
siesta: 84 0.701911 0.325710 0.175703
siesta: 85 -0.264310 -0.250189 0.007174
siesta: 86 -0.175832 0.097385 -0.032617
siesta: 87 -0.331440 -0.122107 -0.042440
siesta: 88 -0.801376 -0.035196 0.041947
siesta: 89 0.033024 -0.367645 0.012942
siesta: 90 -0.702063 -0.326665 0.175003
siesta: 91 0.199651 -0.244242 0.003554
siesta: 92 -0.720223 -0.187568 -0.782983
siesta: 93 0.331187 0.122054 -0.043149
siesta: 94 0.138168 -0.810970 0.054724
siesta: 95 0.167743 -0.151180 0.014744
siesta: 96 0.176607 -0.097026 -0.033053
siesta: 97 0.264518 0.250466 0.007885
siesta: 98 -0.033004 0.367371 0.012229
siesta: 99 0.801046 0.034569 0.042899
siesta: 100 -0.199827 0.244496 0.004278
siesta: --
siesta: Tot 0.005953 -0.001489 -5.526983

siesta: Stress tensor (static) (eV/Ang**3):
siesta: -0.036825 0.000310 0.000001
siesta: 0.001260 -0.039679 0.000001
siesta: 0.000001 0.000001 -0.051221

siesta: Cell volume = 1147.065353 Ang**3

siesta: Pressure (static):
siesta: Solid Molecule Units
siesta: 0.00046370 0.00016372 Ry/Bohr**3
siesta: 0.04257525 0.01503198 eV/Ang**3
siesta: 68.21380431 24.08414675 kBar

$\delta_{\text{filter}} = 10^{-6}$: TC2

siesta: Final energy (eV):

siesta:	Kinetic =	12539.102142
siesta:	Hartree =	9463.951431
siesta:	Ext. field =	0.000000
siesta:	Exch.-corr. =	-5191.559712
siesta:	Ion-electron =	-30280.081607
siesta:	Ion-ion =	-4091.947116
siesta:	Ekinion =	0.000000
siesta:	Total =	-17560.534862

siesta: Atomic forces (eV/Ang):

siesta:	1	0.720433	0.188571	-0.784247
siesta:	2	-0.137346	0.810848	0.052976
siesta:	3	-0.167766	0.151174	0.013170
siesta:	4	0.701837	0.325740	0.174945
siesta:	5	-0.264488	-0.250426	0.006338
siesta:	6	-0.175967	0.096976	-0.033310
siesta:	7	-0.331156	-0.122031	-0.043240
siesta:	8	-0.801344	-0.035035	0.041286
siesta:	9	0.032999	-0.367384	0.011963
siesta:	10	-0.702445	-0.326354	0.174250
siesta:	11	0.199840	-0.244516	0.002713
siesta:	12	-0.720062	-0.188060	-0.783728
siesta:	13	0.331489	0.122148	-0.043948
siesta:	14	0.138544	-0.810926	0.053876
siesta:	15	0.167475	-0.151069	0.013894
siesta:	16	0.176295	-0.096947	-0.033727
siesta:	17	0.264328	0.250223	0.007049
siesta:	18	-0.032949	0.367725	0.011259
siesta:	19	0.801019	0.034294	0.040065
siesta:	20	-0.199654	0.244258	0.003448
siesta:	21	0.720337	0.188796	-0.783413
siesta:	22	-0.137617	0.810800	0.053815
siesta:	23	-0.167495	0.151045	0.013972
siesta:	24	0.701800	0.325753	0.174980
siesta:	25	-0.264278	-0.250204	0.007140
siesta:	26	-0.175973	0.097026	-0.033333
siesta:	27	-0.331172	-0.122044	-0.043187
siesta:	28	-0.801379	-0.035304	0.042086
siesta:	29	0.033019	-0.367399	0.012030
siesta:	30	-0.702153	-0.326531	0.175060
siesta:	31	0.199683	-0.244236	0.003520
siesta:	32	-0.720057	-0.187992	-0.783712

siesta:	33	0.331194	0.122083	-0.043149
siesta:	34	0.138513	-0.810908	0.053922
siesta:	35	0.167490	-0.151058	0.013976
siesta:	36	0.176528	-0.096809	-0.032967
siesta:	37	0.264323	0.250254	0.007110
siesta:	38	-0.032988	0.367424	0.012051
siesta:	39	0.801133	0.034932	0.042241
siesta:	40	-0.199685	0.244249	0.003507
siesta:	41	0.720381	0.188731	-0.783484
siesta:	42	-0.137579	0.810825	0.053771
siesta:	43	-0.167505	0.151052	0.013956
siesta:	44	0.701754	0.325786	0.174942
siesta:	45	-0.264299	-0.250211	0.007123
siesta:	46	-0.175993	0.097085	-0.033430
siesta:	47	-0.331166	-0.122045	-0.043203
siesta:	48	-0.801374	-0.035265	0.041996
siesta:	49	0.033023	-0.367396	0.012024
siesta:	50	-0.702129	-0.326565	0.175002
siesta:	51	0.199682	-0.244246	0.003501
siesta:	52	-0.720074	-0.187933	-0.783739
siesta:	53	0.331203	0.122082	-0.043186
siesta:	54	0.138487	-0.810913	0.053909
siesta:	55	0.167488	-0.151055	0.013961
siesta:	56	0.176537	-0.096895	-0.033076
siesta:	57	0.264323	0.250253	0.007108
siesta:	58	-0.033021	0.367391	0.012173
siesta:	59	0.801124	0.034894	0.042184
siesta:	60	-0.199682	0.244254	0.003500
siesta:	61	0.720396	0.188677	-0.783519
siesta:	62	-0.137563	0.810835	0.053749
siesta:	63	-0.167503	0.151055	0.013957
siesta:	64	0.701696	0.325812	0.174872
siesta:	65	-0.264300	-0.250212	0.007115
siesta:	66	-0.176012	0.097131	-0.033547
siesta:	67	-0.331149	-0.122046	-0.043230
siesta:	68	-0.801362	-0.035228	0.041934
siesta:	69	0.033025	-0.367388	0.012010
siesta:	70	-0.702089	-0.326599	0.174964
siesta:	71	0.199680	-0.244246	0.003494
siesta:	72	-0.720081	-0.187896	-0.783791
siesta:	73	0.331199	0.122078	-0.043200
siesta:	74	0.138480	-0.810914	0.053869
siesta:	75	0.167478	-0.151050	0.013939
siesta:	76	0.176680	-0.097010	-0.033324

```
siesta:     77    0.264313    0.250246    0.007089
siesta:     78   -0.033020    0.367393    0.012162
siesta:     79    0.801100    0.034897    0.042093
siesta:     80   -0.199679    0.244231    0.003483
siesta:     81    0.720458    0.188646   -0.783477
siesta:     82   -0.137548    0.810881    0.053811
siesta:     83   -0.167477    0.151063    0.014011
siesta:     84    0.701912    0.325713    0.175710
siesta:     85   -0.264305   -0.250189    0.007182
siesta:     86   -0.175828    0.097390   -0.032623
siesta:     87   -0.331448   -0.122096   -0.042435
siesta:     88   -0.801378   -0.035192    0.041945
siesta:     89    0.033024   -0.367653    0.012949
siesta:     90   -0.702065   -0.326666    0.174994
siesta:     91    0.199646   -0.244232    0.003556
siesta:     92   -0.720218   -0.187565   -0.782941
siesta:     93    0.331185    0.122056   -0.043124
siesta:     94    0.138164   -0.810974    0.054722
siesta:     95    0.167737   -0.151190    0.014682
siesta:     96    0.176603   -0.097034   -0.033053
siesta:     97    0.264520    0.250464    0.007879
siesta:     98   -0.033008    0.367370    0.012238
siesta:     99    0.801049    0.034567    0.042886
siesta:    100   -0.199833    0.244494    0.004288
siesta: ----------------------------------------
siesta:    Tot    0.005968   -0.001468   -5.526973

siesta: Stress tensor (static) (eV/Ang**3):
siesta:    -0.036825    0.000310    0.000001
siesta:     0.001260   -0.039680    0.000001
siesta:     0.000001    0.000001   -0.051221

siesta: Cell volume =       1147.065353 Ang**3

siesta: Pressure (static):
siesta:                 Solid            Molecule  Units
siesta:            0.00046370          0.00016372  Ry/Bohr**3
siesta:            0.04257526          0.01503200  eV/Ang**3
siesta:           68.21381624         24.08418012  kBar
```

$\delta_{\text{filter}} = 10^{-6}$: DIAG

siesta: Final energy (eV):

siesta:	Kinetic =	12539.102064
siesta:	Hartree =	9463.951289
siesta:	Ext. field =	0.000000
siesta:	Exch.-corr. =	-5191.559678
siesta:	Ion-electron =	-30280.081383
siesta:	Ion-ion =	-4091.947116
siesta:	Ekinion =	0.000000
siesta:	Total =	-17560.534825

siesta: Atomic forces (eV/Ang):

siesta:	1	0.720448	0.188572	-0.784244
siesta:	2	-0.137351	0.810833	0.052976
siesta:	3	-0.167768	0.151177	0.013178
siesta:	4	0.701846	0.325743	0.174947
siesta:	5	-0.264486	-0.250425	0.006338
siesta:	6	-0.175971	0.096975	-0.033310
siesta:	7	-0.331165	-0.122021	-0.043242
siesta:	8	-0.801342	-0.035041	0.041284
siesta:	9	0.032998	-0.367385	0.011965
siesta:	10	-0.702457	-0.326364	0.174245
siesta:	11	0.199832	-0.244503	0.002713
siesta:	12	-0.720065	-0.188065	-0.783740
siesta:	13	0.331491	0.122142	-0.043946
siesta:	14	0.138541	-0.810928	0.053882
siesta:	15	0.167473	-0.151071	0.013905
siesta:	16	0.176294	-0.096961	-0.033724
siesta:	17	0.264330	0.250226	0.007048
siesta:	18	-0.032951	0.367726	0.011252
siesta:	19	0.801023	0.034296	0.040070
siesta:	20	-0.199659	0.244255	0.003440
siesta:	21	0.720335	0.188799	-0.783422
siesta:	22	-0.137617	0.810800	0.053821
siesta:	23	-0.167496	0.151046	0.013980
siesta:	24	0.701804	0.325752	0.174983
siesta:	25	-0.264279	-0.250202	0.007136
siesta:	26	-0.175968	0.097024	-0.033329
siesta:	27	-0.331175	-0.122045	-0.043190
siesta:	28	-0.801378	-0.035308	0.042084
siesta:	29	0.033021	-0.367398	0.012028
siesta:	30	-0.702155	-0.326528	0.175068
siesta:	31	0.199679	-0.244236	0.003518
siesta:	32	-0.720058	-0.187990	-0.783708

siesta:	33	0.331195	0.122082	-0.043152
siesta:	34	0.138513	-0.810908	0.053930
siesta:	35	0.167490	-0.151058	0.013966
siesta:	36	0.176529	-0.096809	-0.032970
siesta:	37	0.264320	0.250252	0.007109
siesta:	38	-0.032988	0.367422	0.012054
siesta:	39	0.801134	0.034934	0.042240
siesta:	40	-0.199684	0.244253	0.003503
siesta:	41	0.720381	0.188733	-0.783489
siesta:	42	-0.137582	0.810825	0.053774
siesta:	43	-0.167504	0.151054	0.013963
siesta:	44	0.701756	0.325785	0.174941
siesta:	45	-0.264295	-0.250210	0.007116
siesta:	46	-0.175992	0.097082	-0.033429
siesta:	47	-0.331169	-0.122046	-0.043206
siesta:	48	-0.801373	-0.035266	0.041997
siesta:	49	0.033022	-0.367395	0.012024
siesta:	50	-0.702128	-0.326568	0.175006
siesta:	51	0.199682	-0.244247	0.003497
siesta:	52	-0.720078	-0.187933	-0.783741
siesta:	53	0.331205	0.122081	-0.043187
siesta:	54	0.138487	-0.810911	0.053911
siesta:	55	0.167485	-0.151056	0.013962
siesta:	56	0.176535	-0.096895	-0.033079
siesta:	57	0.264319	0.250251	0.007104
siesta:	58	-0.033023	0.367391	0.012172
siesta:	59	0.801123	0.034899	0.042182
siesta:	60	-0.199685	0.244252	0.003498
siesta:	61	0.720397	0.188672	-0.783529
siesta:	62	-0.137564	0.810838	0.053754
siesta:	63	-0.167501	0.151053	0.013960
siesta:	64	0.701693	0.325815	0.174879
siesta:	65	-0.264299	-0.250212	0.007111
siesta:	66	-0.176008	0.097133	-0.033547
siesta:	67	-0.331149	-0.122046	-0.043234
siesta:	68	-0.801358	-0.035231	0.041935
siesta:	69	0.033024	-0.367386	0.012010
siesta:	70	-0.702088	-0.326600	0.174964
siesta:	71	0.199681	-0.244247	0.003492
siesta:	72	-0.720083	-0.187893	-0.783801
siesta:	73	0.331199	0.122079	-0.043202
siesta:	74	0.138478	-0.810911	0.053873
siesta:	75	0.167472	-0.151048	0.013947
siesta:	76	0.176678	-0.097010	-0.033318

```
siesta:     77     0.264313     0.250245     0.007085
siesta:     78    -0.033020     0.367392     0.012167
siesta:     79     0.801104     0.034895     0.042095
siesta:     80    -0.199678     0.244235     0.003481
siesta:     81     0.720449     0.188641    -0.783483
siesta:     82    -0.137541     0.810887     0.053815
siesta:     83    -0.167477     0.151060     0.014022
siesta:     84     0.701911     0.325710     0.175703
siesta:     85    -0.264310    -0.250189     0.007174
siesta:     86    -0.175832     0.097385    -0.032617
siesta:     87    -0.331440    -0.122107    -0.042440
siesta:     88    -0.801376    -0.035196     0.041947
siesta:     89     0.033024    -0.367645     0.012942
siesta:     90    -0.702063    -0.326665     0.175003
siesta:     91     0.199651    -0.244242     0.003554
siesta:     92    -0.720223    -0.187568    -0.782983
siesta:     93     0.331187     0.122054    -0.043149
siesta:     94     0.138168    -0.810970     0.054724
siesta:     95     0.167743    -0.151180     0.014744
siesta:     96     0.176607    -0.097026    -0.033053
siesta:     97     0.264518     0.250466     0.007885
siesta:     98    -0.033004     0.367371     0.012229
siesta:     99     0.801046     0.034569     0.042899
siesta:    100    -0.199827     0.244496     0.004278
siesta: ----------------------------------------
siesta:    Tot     0.005953    -0.001489    -5.526983

siesta: Stress tensor (static) (eV/Ang**3):
siesta:    -0.036825     0.000310     0.000001
siesta:     0.001260    -0.039679     0.000001
siesta:     0.000001     0.000001    -0.051221

siesta: Cell volume =       1147.065353 Ang**3

siesta: Pressure (static):
siesta:                Solid           Molecule  Units
siesta:           0.00046370         0.00016372  Ry/Bohr**3
siesta:           0.04257525         0.01503198  eV/Ang**3
siesta:          68.21380431        24.08414675  kBar
```

DZ basis set

$\delta_{\text{filter}} = 10^{-4}$: TC2

siesta: Final energy (eV):

siesta:	Kinetic =	13282.998101
siesta:	Hartree =	10099.502055
siesta:	Ext. field =	0.000000
siesta:	Exch.-corr. =	-5314.203195
siesta:	Ion-electron =	-31601.945851
siesta:	Ion-ion =	-4091.947116
siesta:	Ekinion =	0.000000
siesta:	Total =	-17625.596008

siesta: Atomic forces (eV/Ang):

siesta:	1	0.780283	0.341509	-0.745635
siesta:	2	-0.152623	0.900857	0.160369
siesta:	3	-0.472208	-0.406363	0.063446
siesta:	4	0.697237	0.490362	0.194064
siesta:	5	0.145437	-0.635296	0.016781
siesta:	6	-0.356359	0.177348	-0.096825
siesta:	7	-0.245890	-0.629450	-0.118317
siesta:	8	-0.953411	-0.190991	-0.003265
siesta:	9	0.518258	-0.389013	0.130227
siesta:	10	-0.705948	-0.459617	0.265872
siesta:	11	0.666468	-0.020887	-0.054769
siesta:	12	-0.826749	-0.359687	-0.675474
siesta:	13	0.229051	0.596617	-0.014867
siesta:	14	0.193532	-0.885832	0.068729
siesta:	15	0.517235	0.359493	-0.071997
siesta:	16	0.358350	-0.123421	-0.005652
siesta:	17	-0.180584	0.622677	-0.012841
siesta:	18	-0.679150	0.288199	0.073990
siesta:	19	1.033829	0.169433	0.117807
siesta:	20	-0.674867	-0.001708	-0.027156
siesta:	21	0.809005	0.309820	-0.772253
siesta:	22	-0.218342	0.946504	0.020703
siesta:	23	-0.458011	-0.389741	-0.125038
siesta:	24	0.638734	0.509516	0.283239
siesta:	25	0.224788	-0.729601	-0.071397
siesta:	26	-0.327549	0.163918	0.019628
siesta:	27	-0.182349	-0.593023	-0.109449
siesta:	28	-0.966255	-0.101654	0.064833
siesta:	29	0.576840	-0.381940	-0.000826
siesta:	30	-0.685093	-0.396516	0.223964

siesta:	31	0.704044	-0.023247	0.058895
siesta:	32	-0.834010	-0.332300	-0.815206
siesta:	33	0.271975	0.621109	-0.055009
siesta:	34	0.237721	-0.893678	0.043156
siesta:	35	0.462067	0.405858	-0.096277
siesta:	36	0.297967	-0.165553	-0.102842
siesta:	37	-0.128570	0.609542	0.057605
siesta:	38	-0.606317	0.419948	-0.042766
siesta:	39	0.974618	0.146930	0.058837
siesta:	40	-0.699984	0.043405	-0.010413
siesta:	41	0.784018	0.385583	-0.749752
siesta:	42	-0.257153	0.944228	0.136535
siesta:	43	-0.450141	-0.443117	0.009386
siesta:	44	0.621649	0.477622	0.250616
siesta:	45	0.042350	-0.645937	-0.048195
siesta:	46	-0.347009	0.157540	0.055063
siesta:	47	-0.182869	-0.579467	0.019896
siesta:	48	-0.965161	-0.140698	0.070978
siesta:	49	0.659155	-0.356371	-0.073699
siesta:	50	-0.656535	-0.415978	0.219464
siesta:	51	0.678822	0.015732	-0.064916
siesta:	52	-0.822495	-0.348439	-0.722032
siesta:	53	0.160352	0.599721	-0.085840
siesta:	54	0.238550	-0.904153	0.100543
siesta:	55	0.487690	0.369748	-0.019011
siesta:	56	0.330289	-0.172538	-0.025712
siesta:	57	-0.189853	0.708324	0.057811
siesta:	58	-0.556214	0.388314	0.048860
siesta:	59	0.997342	0.099417	0.084599
siesta:	60	-0.676879	0.079417	-0.011331
siesta:	61	0.755836	0.320421	-0.760767
siesta:	62	-0.229430	0.950354	0.064627
siesta:	63	-0.417722	-0.469741	-0.127548
siesta:	64	0.678609	0.449816	0.252635
siesta:	65	0.234780	-0.653903	-0.018175
siesta:	66	-0.331711	0.148565	-0.032303
siesta:	67	-0.278689	-0.506790	-0.068888
siesta:	68	-1.002687	-0.169264	0.077456
siesta:	69	0.582933	-0.390468	-0.111828
siesta:	70	-0.689823	-0.435762	0.237466
siesta:	71	0.733008	0.006481	-0.062362
siesta:	72	-0.782967	-0.345358	-0.807137
siesta:	73	0.156645	0.624076	-0.036294
siesta:	74	0.255160	-0.914215	-0.002891

siesta:	75	0.489876	0.418648	-0.055047
siesta:	76	0.339096	-0.157362	-0.103349
siesta:	77	-0.208454	0.554119	-0.058192
siesta:	78	-0.605530	0.398463	-0.000616
siesta:	79	0.986770	0.177867	0.090249
siesta:	80	-0.673582	-0.025237	-0.047066
siesta:	81	0.810361	0.331243	-0.779193
siesta:	82	-0.232805	0.963867	0.104773
siesta:	83	-0.462569	-0.329586	0.044186
siesta:	84	0.691719	0.347045	0.218346
siesta:	85	0.231439	-0.578682	-0.018816
siesta:	86	-0.305611	0.137648	0.066582
siesta:	87	-0.226832	-0.645911	-0.220981
siesta:	88	-1.021813	-0.144791	0.020691
siesta:	89	0.633773	-0.469237	0.078310
siesta:	90	-0.691001	-0.468186	0.244425
siesta:	91	0.715812	-0.019842	0.080134
siesta:	92	-0.803716	-0.365804	-0.842988
siesta:	93	0.259406	0.627584	-0.063867
siesta:	94	0.222140	-0.896029	0.040562
siesta:	95	0.427106	0.391050	-0.123621
siesta:	96	0.352590	-0.126340	-0.016951
siesta:	97	-0.146018	0.661254	0.042725
siesta:	98	-0.655721	0.300920	-0.127279
siesta:	99	0.995196	0.139075	0.068722
siesta:	100	-0.658983	0.089898	-0.019326
siesta: --				
siesta:	Tot	0.005667	0.158361	-5.626468

siesta: Stress tensor (static) (eV/Ang**3):

siesta:	-0.005036	-0.001631	-0.000047
siesta:	0.000751	-0.007134	0.000478
siesta:	0.000178	0.000039	0.004567

siesta: Cell volume = 1147.065353 Ang**3

siesta: Pressure (static):

siesta:	Solid	Molecule	Units
siesta:	0.00002760	-0.00003763	Ry/Bohr**3
siesta:	0.00253433	-0.00345519	eV/Ang**3
siesta:	4.06048655	-5.53587652	kBar

$\delta_{\text{filter}} = 10^{-4}$: DIAG

siesta: Final energy (eV):
siesta: Kinetic = 13282.745361
siesta: Hartree = 10099.495174
siesta: Ext. field = 0.000000
siesta: Exch.-corr. = -5314.211678
siesta: Ion-electron = -31601.904233
siesta: Ion-ion = -4091.947116
siesta: Ekinion = 0.000000
siesta: Total = -17625.822492

siesta: Atomic forces (eV/Ang):

siesta:	1	0.797188	0.333828	-0.772220
siesta:	2	-0.224813	0.927828	0.076398
siesta:	3	-0.478220	-0.396861	-0.015444
siesta:	4	0.671111	0.429157	0.190282
siesta:	5	0.183948	-0.635894	-0.018126
siesta:	6	-0.344886	0.144055	-0.012408
siesta:	7	-0.203531	-0.595365	-0.061298
siesta:	8	-0.979055	-0.140362	0.058360
siesta:	9	0.594354	-0.381094	-0.008250
siesta:	10	-0.671676	-0.429822	0.189694
siesta:	11	0.668883	-0.047217	-0.016993
siesta:	12	-0.796793	-0.333327	-0.771822
siesta:	13	0.203726	0.595462	-0.061866
siesta:	14	0.225836	-0.927968	0.077190
siesta:	15	0.477978	0.396883	-0.014855
siesta:	16	0.345127	-0.144098	-0.012729
siesta:	17	-0.184063	0.635713	-0.017550
siesta:	18	-0.594366	0.381259	-0.008823
siesta:	19	0.978854	0.139965	0.058984
siesta:	20	-0.668742	0.047030	-0.016403
siesta:	21	0.797137	0.333989	-0.771453
siesta:	22	-0.225012	0.927828	0.077184
siesta:	23	-0.478018	-0.396938	-0.014780
siesta:	24	0.671058	0.429181	0.190386
siesta:	25	0.184073	-0.635732	-0.017454
siesta:	26	-0.344877	0.144110	-0.012370
siesta:	27	-0.203519	-0.595379	-0.061221
siesta:	28	-0.979112	-0.140566	0.059091
siesta:	29	0.594373	-0.381093	-0.008165
siesta:	30	-0.671443	-0.429989	0.190428
siesta:	31	0.668753	-0.047044	-0.016329
siesta:	32	-0.796800	-0.333266	-0.771726
siesta:	33	0.203517	0.595399	-0.061205

siesta:	34	0.225820	-0.927963	0.077308
siesta:	35	0.477978	0.396900	-0.014770
siesta:	36	0.345328	-0.144024	-0.012012
siesta:	37	-0.184077	0.635730	-0.017468
siesta:	38	-0.594367	0.381042	-0.008157
siesta:	39	0.978844	0.140006	0.059283
siesta:	40	-0.668754	0.047018	-0.016315
siesta:	41	0.797161	0.333928	-0.771489
siesta:	42	-0.224968	0.927853	0.077183
siesta:	43	-0.478018	-0.396936	-0.014773
siesta:	44	0.671011	0.429222	0.190373
siesta:	45	0.184063	-0.635738	-0.017446
siesta:	46	-0.344894	0.144165	-0.012431
siesta:	47	-0.203515	-0.595381	-0.061227
siesta:	48	-0.979100	-0.140509	0.059040
siesta:	49	0.594372	-0.381102	-0.008143
siesta:	50	-0.671404	-0.430033	0.190406
siesta:	51	0.668754	-0.047048	-0.016319
siesta:	52	-0.796823	-0.333218	-0.771737
siesta:	53	0.203517	0.595397	-0.061216
siesta:	54	0.225788	-0.927969	0.077314
siesta:	55	0.477978	0.396899	-0.014765
siesta:	56	0.345339	-0.144104	-0.012091
siesta:	57	-0.184073	0.635730	-0.017466
siesta:	58	-0.594345	0.381069	-0.008238
siesta:	59	0.978833	0.139985	0.059258
siesta:	60	-0.668754	0.047019	-0.016313
siesta:	61	0.797190	0.333863	-0.771490
siesta:	62	-0.224958	0.927869	0.077184
siesta:	63	-0.478013	-0.396935	-0.014770
siesta:	64	0.670937	0.429254	0.190335
siesta:	65	0.184066	-0.635735	-0.017443
siesta:	66	-0.344919	0.144222	-0.012525
siesta:	67	-0.203509	-0.595383	-0.061233
siesta:	68	-0.979091	-0.140480	0.059013
siesta:	69	0.594372	-0.381094	-0.008139
siesta:	70	-0.671364	-0.430058	0.190398
siesta:	71	0.668752	-0.047043	-0.016317
siesta:	72	-0.796835	-0.333174	-0.771766
siesta:	73	0.203515	0.595395	-0.061227
siesta:	74	0.225779	-0.927983	0.077311
siesta:	75	0.477967	0.396903	-0.014761
siesta:	76	0.345434	-0.144198	-0.012245
siesta:	77	-0.184073	0.635725	-0.017465

siesta:	78	-0.594345	0.381067	-0.008237
siesta:	79	0.978813	0.139968	0.059214
siesta:	80	-0.668749	0.047011	-0.016309
siesta:	81	0.797217	0.333825	-0.771407
siesta:	82	-0.224914	0.927891	0.077292
siesta:	83	-0.478008	-0.396924	-0.014679
siesta:	84	0.671093	0.429169	0.191113
siesta:	85	0.184046	-0.635724	-0.017365
siesta:	86	-0.344670	0.144367	-0.011539
siesta:	87	-0.203711	-0.595447	-0.060573
siesta:	88	-0.979120	-0.140427	0.059085
siesta:	89	0.594341	-0.381335	-0.007559
siesta:	90	-0.671311	-0.430113	0.190490
siesta:	91	0.668732	-0.047056	-0.016229
siesta:	92	-0.796917	-0.332897	-0.771020
siesta:	93	0.203417	0.595428	-0.061119
siesta:	94	0.225524	-0.927996	0.078079
siesta:	95	0.478168	0.396826	-0.014092
siesta:	96	0.345320	-0.144194	-0.011842
siesta:	97	-0.183946	0.635894	-0.016798
siesta:	98	-0.594328	0.381060	-0.008152
siesta:	99	0.978746	0.139730	0.059939
siesta:	100	-0.668866	0.047172	-0.015639

siesta: --

siesta:	Tot	0.004465	-0.003945	-5.750189

siesta: Stress tensor (static) (eV/Ang**3):

siesta:	-0.005158	-0.001700	0.000000
siesta:	0.000263	-0.007369	-0.000000
siesta:	0.000000	-0.000000	0.004837

siesta: Cell volume = 1147.065353 Ang**3

siesta: Pressure (static):

siesta:	Solid	Molecule	Units
siesta:	0.00002792	-0.00004789	Ry/Bohr**3
siesta:	0.00256352	-0.00439698	eV/Ang**3
siesta:	4.10726049	-7.04481000	kBarsiesta:

siesta:	0.000000	-0.000000	0.004837

DZP basis set

$\delta_{\text{filter}} = 10^{-4}$: TC2

siesta: Final energy (eV):

siesta:	Kinetic =	13158.588340
siesta:	Hartree =	10141.180511
siesta:	Ext. field =	0.000000
siesta:	Exch.-corr. =	-5311.862941
siesta:	Ion-electron =	-31549.447910
siesta:	Ion-ion =	-4091.947116
siesta:	Ekinion =	0.000000
siesta:	Total =	-17653.489116

siesta: Atomic forces (eV/Ang):

siesta:	1	1.052305	0.515589	-0.581012
siesta:	2	-0.069465	1.433697	0.000147
siesta:	3	-0.248938	-0.017167	0.381301
siesta:	4	0.561418	0.781190	-0.013876
siesta:	5	-0.001143	-0.253913	0.392739
siesta:	6	-0.570558	0.227516	-0.085276
siesta:	7	-0.550036	-0.488722	-0.067147
siesta:	8	-1.257357	-0.251899	0.266049
siesta:	9	0.208330	-0.339617	0.160929
siesta:	10	-0.863287	-0.741504	0.364153
siesta:	11	0.410606	0.007614	0.157837
siesta:	12	-0.837832	-0.668326	-0.740105
siesta:	13	0.122121	0.158310	-0.012331
siesta:	14	0.315292	-1.140566	-0.052669
siesta:	15	0.093472	0.326086	0.067516
siesta:	16	0.574013	-0.341318	0.161712
siesta:	17	0.053902	0.499490	-0.058327
siesta:	18	-0.297674	0.120952	0.040682
siesta:	19	1.352597	0.338441	0.119456
siesta:	20	-0.563222	0.169218	-0.288048
siesta:	21	0.954968	0.551495	-0.838948
siesta:	22	-0.436924	1.159357	0.068006
siesta:	23	-0.034075	-0.303732	0.108693
siesta:	24	0.725035	0.776561	0.301023
siesta:	25	-0.065158	-0.571329	-0.272806
siesta:	26	-0.525511	0.447379	-0.060301
siesta:	27	0.073669	-0.398866	-0.195631
siesta:	28	-1.038824	0.009542	0.195560
siesta:	29	0.114899	-0.363923	-0.047418
siesta:	30	-0.687486	-0.628413	-0.094320

siesta:	31	0.172615	-0.422372	-0.296408
siesta:	32	-0.828868	-0.555311	-0.778823
siesta:	33	0.304212	0.308623	0.099843
siesta:	34	0.296235	-1.156202	0.115377
siesta:	35	0.051748	0.061965	-0.032035
siesta:	36	0.590414	-0.300468	-0.100257
siesta:	37	0.068603	0.342068	0.104361
siesta:	38	-0.404784	0.202507	-0.052619
siesta:	39	1.301646	0.019058	0.088512
siesta:	40	-0.538964	0.330363	0.134846
siesta:	41	0.960877	0.658273	-0.670984
siesta:	42	-0.411302	1.254647	0.097741
siesta:	43	-0.314043	-0.412284	-0.311578
siesta:	44	0.788640	0.651414	0.310198
siesta:	45	0.167805	-0.623515	-0.107979
siesta:	46	-0.589588	0.490629	-0.182037
siesta:	47	-0.161475	-0.202790	0.076573
siesta:	48	-1.176171	0.067476	0.428717
siesta:	49	0.308483	-0.591311	-0.127353
siesta:	50	-0.844301	-0.495400	0.219536
siesta:	51	0.517917	0.065143	-0.247188
siesta:	52	-1.171371	-0.586970	-0.597898
siesta:	53	0.109845	0.403018	-0.204519
siesta:	54	0.311702	-1.297057	0.121492
siesta:	55	0.421901	-0.045614	0.160549
siesta:	56	0.606045	-0.408937	-0.120189
siesta:	57	0.213813	0.697741	0.150883
siesta:	58	-0.545589	0.350166	-0.009393
siesta:	59	1.429108	0.222183	0.114780
siesta:	60	-0.379167	0.044777	-0.056355
siesta:	61	0.896643	0.529140	-0.530805
siesta:	62	-0.212195	1.185097	0.023192
siesta:	63	-0.370258	-0.486468	-0.234169
siesta:	64	0.693485	0.870932	0.337235
siesta:	65	-0.163148	-0.439604	0.114761
siesta:	66	-0.750932	0.007351	-0.122396
siesta:	67	-0.278562	-0.421788	0.029524
siesta:	68	-1.535913	-0.223794	0.237879
siesta:	69	0.569536	-0.030510	-0.397687
siesta:	70	-0.828655	-0.789213	-0.003251
siesta:	71	0.808923	0.041023	0.015819
siesta:	72	-0.916062	-0.536210	-1.049621
siesta:	73	0.310374	0.261715	-0.264289
siesta:	74	0.397083	-1.316197	0.147669

```
siesta:     75    0.279469    0.387452    0.036230
siesta:     76    0.606572   -0.373649    0.104726
siesta:     77    0.006281    0.506388   -0.344952
siesta:     78   -0.459192    0.236858    0.010455
siesta:     79    1.303695   -0.002320    0.053461
siesta:     80   -0.367639    0.334963   -0.266878
siesta:     81    1.043972    0.395370   -0.760870
siesta:     82   -0.388983    1.517206    0.232936
siesta:     83   -0.042520   -0.039745   -0.212023
siesta:     84    0.788707    0.464512    0.208382
siesta:     85    0.045478   -0.255227   -0.271536
siesta:     86   -0.664523    0.218679    0.449070
siesta:     87   -0.164504   -0.659476   -0.100532
siesta:     88   -1.395025   -0.481363    0.143286
siesta:     89    0.379190   -0.214701   -0.254494
siesta:     90   -0.457152   -0.902354    0.211639
siesta:     91    0.301210    0.360829    0.166590
siesta:     92   -0.837287   -0.769279   -0.756442
siesta:     93   -0.197430    0.461971    0.006811
siesta:     94    0.421485   -1.084513    0.263192
siesta:     95    0.163188    0.166418    0.104913
siesta:     96    0.469041   -0.254267    0.179375
siesta:     97    0.164296    0.245032   -0.284108
siesta:     98   -0.288491    0.187367   -0.046291
siesta:     99    1.227475    0.397123    0.147029
siesta:    100   -0.352257    0.240022   -0.069122
siesta: ----------------------------------------
siesta:    Tot    0.026501   -0.180267   -5.039913

siesta: Stress tensor (static) (eV/Ang**3):
siesta:     -0.045344    0.001101   -0.000520
siesta:     -0.001638   -0.048323    0.000549
siesta:     -0.000443    0.000910   -0.075038

siesta: Cell volume =       1147.065353 Ang**3

siesta: Pressure (static):
siesta:                Solid            Molecule  Units
siesta:           0.00061247          0.00025016  Ry/Bohr**3
siesta:           0.05623520          0.02296875  eV/Ang**3
siesta:          90.09969689         36.80038592  kBar
```

$\delta_{\text{filter}} = 10^{-4}$: DIAG

siesta: Kinetic = 13156.724877
siesta: Hartree = 10140.262985
siesta: Ext. field = 0.000000
siesta: Exch.-corr. = -5311.681911
siesta: Ion-electron = -31547.727654
siesta: Ion-ion = -4091.947116
siesta: Ekinion = 0.000000
siesta: Total = -17654.368818

siesta: Atomic forces (eV/Ang):

siesta:	1	1.013414	0.539363	-0.745059
siesta:	2	-0.317582	1.230607	0.102713
siesta:	3	-0.313877	-0.218055	-0.011396
siesta:	4	0.763701	0.772451	0.203144
siesta:	5	0.025273	-0.435605	-0.015920
siesta:	6	-0.629054	0.319339	0.025157
siesta:	7	-0.198349	-0.337343	-0.058531
siesta:	8	-1.331682	-0.161218	0.076170
siesta:	9	0.352473	-0.265135	-0.012897
siesta:	10	-0.764298	-0.773072	0.202554
siesta:	11	0.422881	-0.124957	-0.012160
siesta:	12	-1.013014	-0.538932	-0.744706
siesta:	13	0.198529	0.337447	-0.059076
siesta:	14	0.318519	-1.230807	0.103479
siesta:	15	0.313652	0.218004	-0.010835
siesta:	16	0.629223	-0.319484	0.024828
siesta:	17	-0.025359	0.435417	-0.015377
siesta:	18	-0.352512	0.265291	-0.013424
siesta:	19	1.331505	0.160866	0.077285
siesta:	20	-0.422746	0.124813	-0.011616
siesta:	21	1.013359	0.539513	-0.744318
siesta:	22	-0.317799	1.230613	0.103509
siesta:	23	-0.313695	-0.218087	-0.010728
siesta:	24	0.763648	0.772467	0.203281
siesta:	25	0.025360	-0.435435	-0.015257
siesta:	26	-0.629043	0.319395	0.025235
siesta:	27	-0.198327	-0.337351	-0.058417
siesta:	28	-1.331757	-0.161399	0.076906
siesta:	29	0.352486	-0.265115	-0.012776
siesta:	30	-0.764071	-0.773280	0.203312
siesta:	31	0.422750	-0.124829	-0.011507
siesta:	32	-1.013016	-0.538878	-0.744566
siesta:	33	0.198354	0.337372	-0.058410
siesta:	34	0.318487	-1.230811	0.103645

siesta:	35	0.313646	0.218019	-0.010716
siesta:	36	0.629435	-0.319439	0.025556
siesta:	37	-0.025368	0.435416	-0.015266
siesta:	38	-0.352485	0.265101	-0.012768
siesta:	39	1.331455	0.160743	0.077120
siesta:	40	-0.422752	0.124796	-0.011491
siesta:	41	1.013397	0.539462	-0.744332
siesta:	42	-0.317780	1.230643	0.103537
siesta:	43	-0.313698	-0.218087	-0.010721
siesta:	44	0.763594	0.772500	0.203303
siesta:	45	0.025356	-0.435439	-0.015242
siesta:	46	-0.629073	0.319466	0.025187
siesta:	47	-0.198321	-0.337351	-0.058425
siesta:	48	-1.331734	-0.161348	0.076903
siesta:	49	0.352484	-0.265131	-0.012759
siesta:	50	-0.764041	-0.773319	0.203313
siesta:	51	0.422748	-0.124831	-0.011494
siesta:	52	-1.013050	-0.538825	-0.744534
siesta:	53	0.198353	0.337370	-0.058415
siesta:	54	0.318467	-1.230828	0.103682
siesta:	55	0.313647	0.218016	-0.010714
siesta:	56	0.629430	-0.319517	0.025497
siesta:	57	-0.025362	0.435416	-0.015268
siesta:	58	-0.352458	0.265130	-0.012864
siesta:	59	1.331415	0.160719	0.077113
siesta:	60	-0.422751	0.124792	-0.011489
siesta:	61	1.013421	0.539387	-0.744313
siesta:	62	-0.317764	1.230650	0.103576
siesta:	63	-0.313697	-0.218090	-0.010723
siesta:	64	0.763548	0.772539	0.203305
siesta:	65	0.025353	-0.435439	-0.015248
siesta:	66	-0.629075	0.319512	0.025124
siesta:	67	-0.198321	-0.337353	-0.058429
siesta:	68	-1.331731	-0.161312	0.076898
siesta:	69	0.352483	-0.265131	-0.012753
siesta:	70	-0.763992	-0.773355	0.203335
siesta:	71	0.422743	-0.124830	-0.011502
siesta:	72	-1.013055	-0.538760	-0.744536
siesta:	73	0.198344	0.337368	-0.058433
siesta:	74	0.318469	-1.230849	0.103695
siesta:	75	0.313644	0.218017	-0.010708
siesta:	76	0.629538	-0.319617	0.025373
siesta:	77	-0.025356	0.435414	-0.015264
siesta:	78	-0.352458	0.265124	-0.012870

siesta: 79 1.331406 0.160699 0.077106
siesta: 80 -0.422749 0.124788 -0.011480
siesta: 81 1.013454 0.539340 -0.744178
siesta: 82 -0.317728 1.230686 0.103735
siesta: 83 -0.313697 -0.218076 -0.010594
siesta: 84 0.763677 0.772417 0.204076
siesta: 85 0.025339 -0.435439 -0.015124
siesta: 86 -0.629022 0.319739 0.025654
siesta: 87 -0.198481 -0.337439 -0.057783
siesta: 88 -1.331724 -0.161293 0.077008
siesta: 89 0.352480 -0.265351 -0.012183
siesta: 90 -0.763956 -0.773396 0.203490
siesta: 91 0.422737 -0.124832 -0.011369
siesta: 92 -1.013135 -0.538455 -0.743763
siesta: 93 0.198289 0.337395 -0.058294
siesta: 94 0.318221 -1.230847 0.104482
siesta: 95 0.313825 0.217978 -0.010041
siesta: 96 0.629587 -0.319715 0.025393
siesta: 97 -0.025282 0.435579 -0.014605
siesta: 98 -0.352449 0.265130 -0.012744
siesta: 99 1.331343 0.160437 0.077814
siesta: 100 -0.422879 0.124925 -0.010817
siesta: --
siesta: Tot 0.003337 -0.005814 -4.438735

siesta: Stress tensor (static) (eV/Ang**3):
siesta: -0.044795 0.000217 0.000000
siesta: -0.001107 -0.048401 -0.000000
siesta: 0.000000 -0.000000 -0.071052

siesta: Cell volume = 1147.065353 Ang**3

siesta: Pressure (static):

	Solid	Molecule	Units
siesta:	0.00059629	0.00020086	Ry/Bohr**3
siesta:	0.05474911	0.01844204	eV/Ang**3
siesta:	87.71869957	29.54773123	kBar

$\delta_{\mathrm{filter}} = 10^{-6}$: TC2

siesta: Final energy (eV):

siesta:	Kinetic =	13156.722957
siesta:	Hartree =	10140.263436
siesta:	Ext. field =	0.000000
siesta:	Exch.-corr. =	-5311.681930
siesta:	Ion-electron =	-31547.727222
siesta:	Ion-ion =	-4091.947116
siesta:	Ekinion =	0.000000
siesta:	Total =	-17654.369875

siesta: Atomic forces (eV/Ang):

siesta:	1	1.013495	0.539492	-0.745623
siesta:	2	-0.317753	1.230431	0.103081
siesta:	3	-0.313651	-0.217929	-0.010844
siesta:	4	0.763662	0.772204	0.202900
siesta:	5	0.025210	-0.435377	-0.015997
siesta:	6	-0.628729	0.319358	0.025158
siesta:	7	-0.198289	-0.337538	-0.058549
siesta:	8	-1.331645	-0.161148	0.075949
siesta:	9	0.352255	-0.265179	-0.013214
siesta:	10	-0.764227	-0.773149	0.202592
siesta:	11	0.422834	-0.124879	-0.011941
siesta:	12	-1.012911	-0.539076	-0.744546
siesta:	13	0.198390	0.337452	-0.058344
siesta:	14	0.318462	-1.230779	0.103441
siesta:	15	0.313848	0.217973	-0.010672
siesta:	16	0.629153	-0.319526	0.024817
siesta:	17	-0.025423	0.435392	-0.015635
siesta:	18	-0.352581	0.265300	-0.013435
siesta:	19	1.331467	0.160729	0.077291
siesta:	20	-0.422817	0.124744	-0.011610
siesta:	21	1.013407	0.539395	-0.744089
siesta:	22	-0.318059	1.230480	0.103597
siesta:	23	-0.313526	-0.218116	-0.010959
siesta:	24	0.763697	0.772339	0.203063
siesta:	25	0.025405	-0.435152	-0.015036
siesta:	26	-0.629285	0.319379	0.025356
siesta:	27	-0.198235	-0.337282	-0.058322
siesta:	28	-1.331882	-0.161460	0.077004
siesta:	29	0.352483	-0.264937	-0.012793
siesta:	30	-0.763945	-0.773178	0.203106
siesta:	31	0.423056	-0.124810	-0.011627
siesta:	32	-1.013201	-0.538763	-0.744477
siesta:	33	0.198217	0.337307	-0.058446

siesta:	34	0.318494	-1.230572	0.103651
siesta:	35	0.313569	0.218114	-0.011050
siesta:	36	0.629466	-0.319330	0.025642
siesta:	37	-0.025222	0.435290	-0.015393
siesta:	38	-0.352530	0.265057	-0.012515
siesta:	39	1.331572	0.160640	0.076809
siesta:	40	-0.422971	0.124887	-0.011562
siesta:	41	1.013592	0.539499	-0.744498
siesta:	42	-0.317688	1.230794	0.103672
siesta:	43	-0.313962	-0.217977	-0.010306
siesta:	44	0.763524	0.772405	0.203398
siesta:	45	0.025731	-0.435593	-0.015558
siesta:	46	-0.629233	0.319417	0.025284
siesta:	47	-0.198582	-0.337590	-0.058380
siesta:	48	-1.331680	-0.161362	0.076956
siesta:	49	0.352462	-0.265382	-0.012555
siesta:	50	-0.764051	-0.773049	0.203409
siesta:	51	0.422737	-0.124819	-0.011380
siesta:	52	-1.013112	-0.538748	-0.744529
siesta:	53	0.198448	0.337408	-0.058222
siesta:	54	0.318515	-1.230848	0.103815
siesta:	55	0.313610	0.218091	-0.010773
siesta:	56	0.629413	-0.319437	0.025597
siesta:	57	-0.025055	0.435260	-0.015359
siesta:	58	-0.352300	0.265298	-0.012725
siesta:	59	1.331530	0.160863	0.077040
siesta:	60	-0.422861	0.124909	-0.011531
siesta:	61	1.013616	0.539312	-0.744293
siesta:	62	-0.317679	1.230629	0.103490
siesta:	63	-0.313750	-0.218238	-0.011003
siesta:	64	0.763709	0.772617	0.203448
siesta:	65	0.025281	-0.435633	-0.015249
siesta:	66	-0.629204	0.319407	0.025302
siesta:	67	-0.198332	-0.337392	-0.058431
siesta:	68	-1.331796	-0.161177	0.076817
siesta:	69	0.352512	-0.264980	-0.012942
siesta:	70	-0.763889	-0.773300	0.203182
siesta:	71	0.422979	-0.124926	-0.011526
siesta:	72	-1.012837	-0.538709	-0.744509
siesta:	73	0.198223	0.337402	-0.058201
siesta:	74	0.318301	-1.231048	0.103805
siesta:	75	0.313487	0.217647	-0.010799
siesta:	76	0.629501	-0.319621	0.025126
siesta:	77	-0.025619	0.435585	-0.015241

```
siesta:     78   -0.352525    0.264926   -0.012796
siesta:     79    1.331539    0.160848    0.077188
siesta:     80   -0.422973    0.124763   -0.011569
siesta:     81    1.013544    0.539431   -0.744160
siesta:     82   -0.317796    1.230787    0.103798
siesta:     83   -0.313843   -0.218186   -0.010302
siesta:     84    0.763461    0.772290    0.203621
siesta:     85    0.025846   -0.435552   -0.015093
siesta:     86   -0.628966    0.319702    0.025766
siesta:     87   -0.198492   -0.337330   -0.057894
siesta:     88   -1.331845   -0.161308    0.077019
siesta:     89    0.352463   -0.265367   -0.012842
siesta:     90   -0.763955   -0.773426    0.203211
siesta:     91    0.422521   -0.124849   -0.011384
siesta:     92   -1.013189   -0.538795   -0.743699
siesta:     93    0.198318    0.337604   -0.058400
siesta:     94    0.318180   -1.230852    0.104959
siesta:     95    0.313778    0.218127   -0.010316
siesta:     96    0.629896   -0.319708    0.025569
siesta:     97   -0.025232    0.435698   -0.014413
siesta:     98   -0.352399    0.265333   -0.012708
siesta:     99    1.331310    0.160668    0.077901
siesta:    100   -0.422794    0.125110   -0.010452
siesta: ----------------------------------------
siesta:    Tot    0.003649   -0.005586   -4.437888

siesta: Stress tensor (static) (eV/Ang**3):
siesta:     -0.044795    0.000218   -0.000000
siesta:     -0.001107   -0.048399   -0.000000
siesta:      0.000001   -0.000001   -0.071051

siesta: Cell volume =       1147.065353 Ang**3

siesta: Pressure (static):
siesta:                Solid            Molecule  Units
siesta:           0.00059628          0.00020087  Ry/Bohr**3
siesta:           0.05474823          0.01844336  eV/Ang**3
siesta:          87.71728649         29.54984541  kBar
```

$\delta_{\text{filter}} = 10^{-6}$: DIAG

siesta: Final energy (eV):

siesta:	Kinetic =	13156.724877
siesta:	Hartree =	10140.262985
siesta:	Ext. field =	0.000000
siesta:	Exch.-corr. =	-5311.681911
siesta:	Ion-electron =	-31547.727654
siesta:	Ion-ion =	-4091.947116
siesta:	Ekinion =	0.000000
siesta:	Total =	-17654.368818

siesta: Atomic forces (eV/Ang):

siesta:	1	1.013414	0.539363	-0.745059
siesta:	2	-0.317582	1.230607	0.102713
siesta:	3	-0.313877	-0.218055	-0.011396
siesta:	4	0.763701	0.772451	0.203144
siesta:	5	0.025273	-0.435605	-0.015920
siesta:	6	-0.629054	0.319339	0.025157
siesta:	7	-0.198349	-0.337343	-0.058531
siesta:	8	-1.331682	-0.161218	0.076170
siesta:	9	0.352473	-0.265135	-0.012897
siesta:	10	-0.764298	-0.773072	0.202554
siesta:	11	0.422881	-0.124957	-0.012160
siesta:	12	-1.013014	-0.538932	-0.744706
siesta:	13	0.198529	0.337447	-0.059076
siesta:	14	0.318519	-1.230807	0.103479
siesta:	15	0.313652	0.218004	-0.010835
siesta:	16	0.629223	-0.319484	0.024828
siesta:	17	-0.025359	0.435417	-0.015377
siesta:	18	-0.352512	0.265291	-0.013424
siesta:	19	1.331505	0.160866	0.077285
siesta:	20	-0.422746	0.124813	-0.011616
siesta:	21	1.013359	0.539513	-0.744318
siesta:	22	-0.317799	1.230613	0.103509
siesta:	23	-0.313695	-0.218087	-0.010728
siesta:	24	0.763648	0.772467	0.203281
siesta:	25	0.025360	-0.435435	-0.015257
siesta:	26	-0.629043	0.319395	0.025235
siesta:	27	-0.198327	-0.337351	-0.058417
siesta:	28	-1.331757	-0.161399	0.076906
siesta:	29	0.352486	-0.265115	-0.012776
siesta:	30	-0.764071	-0.773280	0.203312
siesta:	31	0.422750	-0.124829	-0.011507
siesta:	32	-1.013016	-0.538878	-0.744566
siesta:	33	0.198354	0.337372	-0.058410

siesta:	34	0.318487	-1.230811	0.103645
siesta:	35	0.313646	0.218019	-0.010716
siesta:	36	0.629435	-0.319439	0.025556
siesta:	37	-0.025368	0.435416	-0.015266
siesta:	38	-0.352485	0.265101	-0.012768
siesta:	39	1.331455	0.160743	0.077120
siesta:	40	-0.422752	0.124796	-0.011491
siesta:	41	1.013397	0.539462	-0.744332
siesta:	42	-0.317780	1.230643	0.103537
siesta:	43	-0.313698	-0.218087	-0.010721
siesta:	44	0.763594	0.772500	0.203303
siesta:	45	0.025356	-0.435439	-0.015242
siesta:	46	-0.629073	0.319466	0.025187
siesta:	47	-0.198321	-0.337351	-0.058425
siesta:	48	-1.331734	-0.161348	0.076903
siesta:	49	0.352484	-0.265131	-0.012759
siesta:	50	-0.764041	-0.773319	0.203313
siesta:	51	0.422748	-0.124831	-0.011494
siesta:	52	-1.013050	-0.538825	-0.744534
siesta:	53	0.198353	0.337370	-0.058415
siesta:	54	0.318467	-1.230828	0.103682
siesta:	55	0.313647	0.218016	-0.010714
siesta:	56	0.629430	-0.319517	0.025497
siesta:	57	-0.025362	0.435416	-0.015268
siesta:	58	-0.352458	0.265130	-0.012864
siesta:	59	1.331415	0.160719	0.077113
siesta:	60	-0.422751	0.124792	-0.011489
siesta:	61	1.013421	0.539387	-0.744313
siesta:	62	-0.317764	1.230650	0.103576
siesta:	63	-0.313697	-0.218090	-0.010723
siesta:	64	0.763548	0.772539	0.203305
siesta:	65	0.025353	-0.435439	-0.015248
siesta:	66	-0.629075	0.319512	0.025124
siesta:	67	-0.198321	-0.337353	-0.058429
siesta:	68	-1.331731	-0.161312	0.076898
siesta:	69	0.352483	-0.265131	-0.012753
siesta:	70	-0.763992	-0.773355	0.203335
siesta:	71	0.422743	-0.124830	-0.011502
siesta:	72	-1.013055	-0.538760	-0.744536
siesta:	73	0.198344	0.337368	-0.058433
siesta:	74	0.318469	-1.230849	0.103695
siesta:	75	0.313644	0.218017	-0.010708
siesta:	76	0.629538	-0.319617	0.025373
siesta:	77	-0.025356	0.435414	-0.015264

siesta:	78	-0.352458	0.265124	-0.012870
siesta:	79	1.331406	0.160699	0.077106
siesta:	80	-0.422749	0.124788	-0.011480
siesta:	81	1.013454	0.539340	-0.744178
siesta:	82	-0.317728	1.230686	0.103735
siesta:	83	-0.313697	-0.218076	-0.010594
siesta:	84	0.763677	0.772417	0.204076
siesta:	85	0.025339	-0.435439	-0.015124
siesta:	86	-0.629022	0.319739	0.025654
siesta:	87	-0.198481	-0.337439	-0.057783
siesta:	88	-1.331724	-0.161293	0.077008
siesta:	89	0.352480	-0.265351	-0.012183
siesta:	90	-0.763956	-0.773396	0.203490
siesta:	91	0.422737	-0.124832	-0.011369
siesta:	92	-1.013135	-0.538455	-0.743763
siesta:	93	0.198289	0.337395	-0.058294
siesta:	94	0.318221	-1.230847	0.104482
siesta:	95	0.313825	0.217978	-0.010041
siesta:	96	0.629587	-0.319715	0.025393
siesta:	97	-0.025282	0.435579	-0.014605
siesta:	98	-0.352449	0.265130	-0.012744
siesta:	99	1.331343	0.160437	0.077814
siesta:	100	-0.422879	0.124925	-0.010817

siesta: --

siesta: Tot 0.003337 -0.005814 -4.438735

siesta: Stress tensor (static) (eV/Ang**3):

siesta:	-0.044795	0.000217	0.000000
siesta:	-0.001107	-0.048401	-0.000000
siesta:	0.000000	-0.000000	-0.071052

siesta: Cell volume = 1147.065353 Ang**3

siesta: Pressure (static):

siesta:	Solid	Molecule	Units
siesta:	0.00059629	0.00020086	Ry/Bohr**3
siesta:	0.05474911	0.01844204	eV/Ang**3
siesta:	87.71869957	29.54773123	kBar
